# De Novo Variants Predominate in Autism Spectrum Disorder

**DOI:** 10.3390/genes16091099

**Published:** 2025-09-17

**Authors:** Richard G. Boles, Omri Bar, Philip T. Boles, Zoë R. Hill, Richard E. Frye

**Affiliations:** 1 Mitochondrial & Molecular Medicine, Pasadena, CA 91108, USA; omribar12@gmail.com (O.B.); philiptboles@gmail.com (P.T.B.); 2 NeuroNeeds^®^, Old Lyme, CT 06371, USA; 3 Autism Discovery and Treatment Foundation, Phoenix, AZ 85050, USA; zoe.hill@autismdiscovery.org (Z.R.H.); drfrye@autismdiscovery.org (R.E.F.)

**Keywords:** autism, disease model, diagnostic yield, DNA sequencing, missense, polygenic inheritance, silent variants, synonymous variants, whole genome sequencing

## Abstract

Background: Autism spectrum disorder (ASD) is a common condition with substantial personal and financial burdens of lifelong implication. Multiple twin studies have confirmed a genetic or inherited component at ~80%, higher than any other common condition. However, ASD’s rapidly accelerating prevalence, now at 1 in 31 in the USA, appears to defy a predominantly genetic basis and implicate our rapidly changing environment. A potential explanation for this paradox is a recent increase in de novo variants (DNVs), which are “new” mutations present in the patient yet absent in both parents. The present authors recently reported using trio whole-genome sequencing (trio-WGS) that DNVs highly likely to be highly disease-associated (“Principal Diagnostic Variants”, PDVs), mostly missense variants, were present in (25/50) 50% of the ASD patients clinically evaluated by our team. Methods: The current study was designed to support this observation with trio-WGS in 100 additional unrelated ASD patients. Results: De novo PDVs were identified in 47/100 (47%) of cases, in close approximation to our previous work. Using non-transcribed (up and downstream) variants for all genes as a control group, these DNV-PDVs were far more likely (*p* < 0.0001, OR 5.8, 95% C.I. 2.9–11) to be in SFARI-listed genes associated with ASD. Consistent with the emerging polygenic model, using the same analyses, inherited missense variants were also associated with ASD (*p* < 0.0001). Highly unexpectedly, silent variants, both inherited (*p* < 0.0001) and de novo (*p* < 0.007), were also statistically associated with ASD, and, among inherited variants, silent variants were more associated with ASD than were missense variants (*p* < 0.0001). Adding silent DNVs as PDVs increases the proportion of our subjects with at least one DNV-PDV to 55% of the subjects. Conclusions: Our proposed model for ASD, with prominent DNVs in most that are genetic yet not inherited, predicts the known predominant genetic pathogenesis and the accelerating prevalence of ASD, possibly from environmental factors, including insufficient nutrients and toxicant exposures, and/or the disrupted folate metabolism known to be associated with ASD. Limitations to this study include predominant inclusion of severely affected individuals and the lack of an unaffected control group and functional validation of variant pathogenicity.

## 1. Introduction

Autism spectrum disorder (ASD) is a neurodevelopmental disorder present in early life with a core dual deficit in social communication and repetitive and/or restricted interests or behaviors [1]. Diagnosis is currently based on the observable behavioral phenotype with little convincing evidence of consistent biomarkers, suggesting biological heterogeneity [2]. Multiple studies have demonstrated that the heritability component in ASD is about 80% [3,4,5], which is the highest reported among all common (prevalence > 1%) disorders. However, frequent episodes of acute/sub-acute onset of severe ASD symptoms following environmental physiological stressors (e.g., infections) suggest the addition of critical environmental components [6]. Thus, in terms of pathogenesis, ASD is oftentimes similar to other common disorders (e.g., diabetes, asthma), in that there are underlying genetic factors resulting in biological vulnerability and environmental factors that may trigger disease onset or exacerbation.

ASD has also increased dramatically in incidence and prevalence, being a rare disorder (<1 in 1000) only 30 years ago, yet most recently found to be present in 1 out of 31 American children [7], which translates to over 10 million currently affected individuals in the USA. Some sources state that the explosion in ASD incidence is a result of better recognition and diagnosis [8]. However, a prevailing view of many of us “in the trenches” (e.g., pediatricians, educators) is that we were not seeing this magnitude of affected children previously under any label, be that intellectual disability, learning disability, or psychiatric disease [9], and that the accelerating disease increase is real. ASD results in great personal costs to the individuals affected and their family members, at least for the more severely affected among the vast spectrum of severity. The economic and societal burden of ASD is substantial, with lifetime care costs exceeding USD 2.4 million per individual (Autism Speaks, 2025) [8]. This provides an estimated, lifetime national cost of USD 25 trillion, very near the amount of the publicly held US national debt. This estimate presumes that the incidence of ASD will not continue to increase, a major assumption that unfortunately may not come to pass.

While the pathogeneses of most common disorders have recently given way to advances in the biological sciences, and this knowledge has generally resulted in improved clinical outcomes, ASD has been somewhat of a hold out. Given the overwhelming burden of the disease, why do we not better understand the pathogenesis of ASD? One of the main reasons is the extreme genotypic heterogeneity of ASD, which includes several hundred genes already so identified [10,11]. As genetic studies in ASD routinely identify multiple additional disease-associated genes, it appears that we have only identified a small minority of those genes to date, and that there are likely thousands of genes associated with ASD. In multiplex families (with two or more ASD-affected first-degree relatives), marked variable expressivity is common among the affected relatives, both in terms of disease severity and the presence and type of comorbid disease manifestations. In many cases, close relatives of people with ASD are themselves affected with another neurodevelopmental disorder or a forme fruste (incomplete or mild) phenotype (e.g., attention deficit hyperactivity disorder (ADHD), learning disabilities). Marked variable expressivity and forme fruste phenotypes are common even when a specific gene variant segregating in a family meets multiple criteria to be considered as a principal cause of disease [12], and this is highly suggestive of polygenic factors (e.g., genetic modifiers, genetic background) and/or environmental factors.

The key role of rare, highly penetrant variants (disease is likely in the presence of the variant), either inherited or non-inherited (de novo), in the development of ASD has been established by many studies (reviewed in [13,14]). Highly penetrant variants predisposing toward ASD often can be identified using DNA sequencing, with inheritance patterns revealed being either Mendelian (e.g., autosomal recessive or dominant, X-linked) or non-Mendelian (e.g., polygenic, maternal, de novo). Recent papers have tended to focus on de novo variants (DNVs, new mutations, absent from both parents) as being of particular importance in ASD. In recent years, these variants are often identified by whole-genome sequencing (WGS, covering over 99% of the entire DNA) in samples collected from trios (affected individual plus biological parents).

The yield of DNVs in ASD has been measured in trio-WGS studies at 20% [15], 21% [16], 31% [17], 41% [18], and 50% [14]. The methodology varied somewhat among these studies, although in general they did not comprehensively query for genes not previously identified in ASD. A study on 50 consecutive, unrelated ASD trios from the practice of the first author (RGB) [14] revealed a DNV diagnostic yield of 20%, if based solely on DNVs listed in the official report from the commercial laboratory (Variantyx, Framingham, MA, USA). However, the diagnostic yield for a de novo Principal Diagnostic Variant (PDV) was 50% (25/50) when trio-WGS was followed by comprehensive reanalysis of the raw DNA sequence data [14]. We defined a PDV using strict criteria (see the Subjects and Methods section) to ensure that each variant so designated is highly likely to be disease-related in that patient, and not an incidental finding. Of interest, the vast majority (15/18, 83%) of all DNVs not listed in the official laboratory report were in genes not previously reported in ASD (13 DNVs) or in those reported only in one to four individuals each (2 DNVs), and thus not expected to be listed in the report by any commercial diagnostic laboratory. This highlights both the likelihood that only a minority of ASD genes have so far been identified and the need to go beyond the commercial laboratory report in ASD diagnostics.

How does the autism community reconcile the conundrum of a disorder that is highly genetic in etiology with its rapidly expanding prevalence? For the most part, it does not, with various aspects of the community denying either the genetic basis or a truly expanding incidence. Assuming that both statements are correct, how can a genetic disorder increase rapidly in the population? One possible explanation involves DNVs, which are genetic but not inherited, with the DNVs themselves accelerated over time by environmental, likely chemical, mutagenesis, nutritional insufficiencies, or the known folate metabolism abnormalities associated with ASD. In the current study, we expand on our previous study to analyze trio-WGS data from Variantyx in 100 consecutive, unrelated subjects with ASD from the practice of the senior author (REF), with a focus on DNVs. Our data confirm earlier studies that DNVs are a major component of the genetic predisposition toward ASD and that they can be identified by trio-WGS followed by raw-data analysis. Is this an answer to the conundrum?

## 2. Subjects and Methods

### 2.1. Subjects

Our subjects consist of the 100 most recently evaluated, sequential, unrelated patients with a clinical diagnosis of ASD in which trio-WGS was performed at Variantyx^®^ (Framingham, MA, USA). Each subject was evaluated clinically by the senior author, who is a child neurologist known for conducting clinical care and research in ASD. At a minimum, the evaluation in all subjects included a detailed history and a physical examination, either in person or via video-teleconferencing. The diagnosis of ASD in each case was confirmed by appropriate neuropsychiatric testing (e.g., ADOS-2). Subjects with additional neurodevelopmental diseases (NDDs) or non-NDD diagnoses were not excluded. In the few cases where more than one family member met the study criteria, the subject was assigned to be the proband (person first presenting as a patient). In cases of affected siblings presenting simultaneously, the elder was assigned. Thus, all study subjects have no known genetic relationships with each other. This study was approved by the Advarra institutional review board (IRB, human subjects committee, cirbi@advarra.com) as a retrospective chart review of available clinical records. No additional testing was performed for the purpose of this study. Trio-WGS in our 100 subjects was performed from January 2022 to July 2024, with our analysis of the raw individual sequence data completed from January through July 2024.

### 2.2. Sequencing and Data Analysis

Available clinical notes from all subjects were reviewed for phenotypic data. WGS analyses from Variantyx^®^ included genome-wide sequence analysis (for single-nucleotide variants and small deletions/insertions), genome-wide structural variant analysis (for copy number variants (CNVs), including large duplications/deletions/inversions, mobile inserions, and aneuploidy), and mitochondrial genome sequence analysis (for heteroplasmy ≥ 5% and large deletion analysis). See our previous study for details of our DNA sequence data analysis, including Figure 1 of that paper for the Variantyx analysis pipeline [14]. Additional information is available at variantyx.com [19]. Raw genomic data from each subject were evaluated on the Variantyx^®^ bioinformatics platform accessible to laboratory personnel in order to tabulate all de novo variants predicted to alter the amino acid code of any protein (“coding” variants). Analyses included the Integrative Genomics Viewer (IGV) of all small de novo variants and SVPlots of all large de novo variants to verify the presence of that variant and exclude artifacts. Inherited sequence variants were tabulated by the same software. In order to compare only protein-coding genes among the various variant types, all non-coding genes were manually removed, including RNA genes (e.g., gene symbols starting with LCA, LINC, LINP, LNC, Metazoa, MIR, MIRNA, PIRNA, PIWIL, RN7, RNA, RNU, RNV, RPL, SNOR, SNRNA, TRNA, U#, or YRNA), antisense genes (gene symbols ending with AS#), and pseudogenes (gene symbols that end with P#), where # is any number. These analyses were conducted genome-wide (on all genes) and were highly laborious; thus, they were completed only on a randomized subset of 50 subjects (25 for non-transcribed variants). Intronic variants were not tabulated as they are extremely numerous and continuously resulted in error messages from the Variantyx software.

### 2.3. Gene Categorization

In the determination of diagnostic yield, we sought to be conservative in that each variant determined to be disease-causal (PDVs) has a high probability of being so. In our previous study [14], we restricted PDV annotation to genes published with direct association with ASD, designated as A1 (the highest direct association) through A3 (the lowest direct association), in particular using SFARI rankings [11] as per Table 5 in our previous work [14]. Genes without a direct association with ASD were designated as B1 (indirect association) through B3 (highly unlikely to be ASD-associated). With the understanding that our prior B1 category was too broad, in the present study, we designated those genes with a published, one-degree-indirect, association to ASD as B0. This category contains genes with a direct association with other conditions associated with ASD (e.g., AD/HD, intellectual disability, schizophrenia, bipolar) and genes with a direct association with another gene that is itself directly related (A1–3) to ASD. The remainder of the prior B1 category comprises our current B1 category. Overall, B1–3 genes are most likely not associated with ASD, but association cannot be excluded.

### 2.4. Variant Categorization

In essence, we used standard American College of Medical Genetics and Genomics (ACMGG) criteria with some modifications designed to allow these criteria to apply to novel disorders. Details on the modifications are listed below, discussed in our previous study [14], including a figure delineating the Variantyx variant annotation pipeline, and further elaborated upon in the Discussion section. Variants were assigned as PDVs if they are real (verified using IGV or SVPlots), coding (changing the amino acid code), rare (allelic prevalence < 1/10,000, population prevalence < ~2/10,000), and evolutionarily conserved (at least moderate, conserved through mammals) in a gene published as directly (A1–A3) or single-step-indirectly (B0) associated with ASD. De novo mitochondrial DNA (mtDNA) variants were eligible for PDV status if coding and the subject had ≥40% and ≥2 times the heteroplasmic % as the mother (a presumed de novo event in the grandmother). Large deletions were counted as evolutionarily conserved when any conserved nucleotide was deleted. Characteristics of different types of coding variants (e.g., missense, frameshift, deletion), and the importance of prevalence and conservation to variant annotation, can be found in a recent review (Tables 2–4 of [20]). Moderate conservation was assumed present if both PhyloP and PhastCons were >0.7 and assumed absent if both were <0.4. Otherwise, conservation was manually determined using the University of California Santa Cruz (UCSC) Genome Browser [21] using a threshold of 80% of listed mammalian species. Splice-site variants were included if >0.6 on SpliceRF or SpliceADA. Thus, the focus of this study was on rare, high-penetrance variants. Statistical analyses were performed using a two-tailed Fisher Exact Test [22] and/or MedCalc^®^ odds ratio calculator [23]. Based on our data analysis, silent DNVs were reclassified as PDVs (see the Results and Discussion sections). Note that CNVs widely considered to be Pathogenic/disease-associated were designated as PDVs regardless of other parameters. Those DNVs that met our criteria for being highly likely to be a genetic factor in that individual’s predisposition to develop ASD we refer to by a separate name (“Principal Diagnostic Variant” (PDV)) instead of “Pathogenic” or “Likely Pathogenic” to underscore that modifications were made from the dominant ACMGG criteria.

## 3. Results

### 3.1. Subject Characteristics

Among our 100 unrelated subjects, the age at the time of sequence review ranged from 4 to 40 years, with a median of 9 years. Mean maternal and paternal ages at the subject’s birth were 33.1 and 35.4 years, respectively. The race of 23 subjects was not recorded. Among the 77 subjects whose race or ethnicity were recorded, 43/77 (56%) were Caucasian, 30/77 (39%) were of other backgrounds (14 South Asians, 3 East Asians, 4 African Americans, and 9 Latinos), and 4/77 (5%) subjects were of mixed race or ethnicity. Twenty subjects (20%) were female. Intellectual disability (ID) was moderate or more in severity in 85/95 (89%) subjects. Twenty-nine (29%) subjects were nonverbal; 31 (31%) had epilepsy; and 57 (57%) experienced at least one episode of substantial developmental regression. Nine (9%) had tics, a potential sign of autoimmune encephalopathy. Additional clinical information is shown in Table S1.

### 3.2. De Novo Variants Identified and Their Characteristics

A total of 151 de novo variants (DNVs) were identified genome-wide that alter the amino acid code of any protein among the 100 subjects (mean 1.5 per subject, range 0–6, Table 1). Among these 151 DNVs, only 17 (present in 15 of the 100 subjects) were reported in the Variantyx laboratory report, and all 17 met the criteria for Principal Diagnostic Variants (PDVs) by our algorithm (Table 2, light blue background in column 1). Only 6 of those 17 variants (in six different subjects) were reported as “Positive” (Pathogenic, determined to be highly likely to be disease-related/causal) by the laboratory, and half (3) of those were large CNV deletions. Adding in an additional nine DNVs (in 7 subjects) with indeterminate designations by the laboratory (labeled as “Other variants of interest”, “Uncertain”, or “Supplementary”), the yield of genetic testing for DNVs related to disease in our cohort was 15/100 (15%). Two additional laboratory-report-listed DNVs labeled as “Negative” and “Likely Negative” were not counted, but if they are counted the yield increases to 17%. Note that this is not the “laboratory yield” as this analysis is limited to DNVs, and there were subjects with results indicating positive laboratory results for inherited variants.

Following our comprehensive sequence reanalysis (as per the Subjects and Methods section and [14]), we identified an additional 41 DNVs as PDVs. At least one DNV-PDV was identified in 47/100 subjects (47%). After adding the additional 19 silent DNVs we identified (based upon our analyses discussed later in this section), a total of 79 DNVs met our criteria for PDVs (Table 2, yellow background in column 1), with at least one DNV-PDV in 55 subjects. Thus, the overall yield for having at least one DNV labeled as a PDV was 55/100 (55%) subjects. Among the 79 DNV-PDVs, there were 43 missense (one on the X chromosome), 19 silent (one on the mtDNA), 4 frameshift, 3 nonsense (stop codon gain), 2 splice site, and 7 large copy number variants (4 duplications and 3 deletions). One, two, three, and four PDVs were identified in 37, 15, 2, and 1 subject(s), respectively (Table 1).

An additional 48 DNVs (none listed in the laboratory reports) were excluded as PDVs (17 for no/inadequate published link to ASD (B1–3 genes), 8 for inadequate evolutionary conservation, 10 for both gene association and conservation, 6 for being in genes in which autosomal recessive (AR) inheritance is well-established, but not autosomal dominant inheritance (likely indicating carrier status), 2 for AR plus gene association, 2 for AR, gene, and conservation, and 1 for prevalence). Regarding the latter, variants below an allelic prevalence of > 1/10,000 were excluded by the computer software, but one borderline case was manually excluded from our analyses (yet shown in Table 1). In subject 32, we labeled one DNV as a PDV in the *KDM5B* gene, which is known to have autosomal recessive inheritance, because of the presence of an additional inherited, rare, and highly conserved missense variant, although the phase is unknown.

A total of 73 DNV-PDVs involving only a single gene (72 single-nucleotide variants (SNVs) and one smaller CNV) were identified in 50 subjects. Among these 73 DNVs, 30 (41%) were in genes with ≥10 individual cases reported with clinical phenotypes (see Table 2 legend, #4 for details), and, thus, were labeled as “Known” disorders (Table 2, column 4). Another 19 DNV-PDVs (26%) were in genes with one to nine cases so reported, and, thus, labeled as “Very rare” disorders. Finally, 24 (33%) were in genes without any cases so reported, and, thus, labeled as “Novel” disorders. With the sole exception of two subjects (49 and 50) with DNV-PDVs in the titan (*TTN*) gene, a Known disorder, every gene is listed only once. Among the 24 Novel disorders, 10 have at least one case listed on the Human Genome Mutation Database (HGMD). Most of these HGMD listings are indexed from studies where over 1000 individuals were sequenced, and no phenotypic (if ASD criteria were truly met) or genotypic (variant parameters differentiating apparent pathogenic from benign) details are available. Thus, other cases may have been identified, although this is unclear in the absence of published phenotypic or genotypic elaboration. Fourteen of the Novel disorders have no case reports and no HGMD listings. For all 24 Novel disorders, the information in the present Table S1 (phenotypic), Table 1 (genotypic), and Table 2 (putative mechanistic) constitute the first true report.

Comparison of clinical data with the presence or absence of a DNV-PDV is fraught with low numbers for many parameters. However, finding such a variant was statistically more likely in the vast majority of the subjects with at least moderate intellectual disability (51/85, 60% versus 2/10, 20%, *p* = 0.02). There were trends for an increased likelihood of identifying a DNV-PDV in those more clinically affected regarding verbal ability (20/29, 69% versus 35/71, 49%, *p* = 0.08), epilepsy (18/36, 50% versus 13/36, 36%, *p* = 0.3), and a history of regression (36/60, 60% versus 19/40, 48%, *p* = 0.2). There were no significant differences or trends for adult age (>18 versus <18 years, *p* = 1.0), female sex (*p* = 0.8), or the presence of tics (*p* = 0.7).

### 3.3. Protein Functions and Pathways Related to the Identified DNV-PDVs

A synopsis of the known functions of each protein encoded by a DNV-PDV is shown in Table 2. Among the 73 of these variants involving only a single gene, known functions were tabulated for selected pathways (ion transport (13 PDVs, red in in Table 2), mitochondrial redox potential/energy metabolism/cell death responses (5, orange), immune system manifestations (11, yellow), ubiquitin-related protein degradation pathway (5, green), synapse/neurotransmission-related (12, blue), gene expression (17, purple), neurogenesis/brain development (14, pink), cytoskeleton-related (10, light grey) cell–cell interactions including adhesion (6, dark grey), signaling pathways other than synaptic transmission (14, black), and cell danger responses (5, brown)). As the causal gene is unclear for CNVs encompassing more than one gene, these data are not included in the above numbers, but the pathways related to the best candidate genes are shown in Table 2.

### 3.4. Tallying Inherited and De Novo Variants in Our Subjects

As ASD is often considered to be polygenic even within affected individuals [14,24], the total number of inherited variants, among all 20,000–23,000 genes, was tallied in a randomized group of our subjects for specific variant types (missense, silent, UTR (untranslated regions 5′ and 3′ added together), and up/downstream (~1 kb adjacent to each gene in each direction, added together)) and compared to de novo missense and silent variants (Table 3). The average number per subject for each variant type is shown in row 5 of Table 3. Each variant was queried as to whether the gene is listed in SFARI or not, and the average number of variants in SFARI genes for each variant type per subject is shown in row 7.

As shown in column B, among our 100 subjects, 11 of 43 (27%) small (not CNV), nuclear (not mtDNA), missense de novo PDVs are present in SFARI-listed genes. Among the same subjects (column D), 696 of 7838 (9.0%) inherited, small, nuclear, missense variants, genome-wide, are present in SFARI-listed genes (*p* = 0.0004, odds ratio 3.4, 95% confidence interval (CI) 1.7–6.8; cell B11, yellow background). Thus, de novo PDV missense variants are about 3½ times more likely to be SFARI-listed than are inherited missense variants among our subjects.

While non-transcribed variants in the vicinity of the gene (e.g., up/downstream) can affect protein function, it is widely believed that the vast majority of them do not, and thus these variants were chosen to be our controls. Being conservative and estimating the total number of genes adequately sequenced by Variantyx to be 20,000, the 1114 ASD-related genes listed by SFARI comprise 5.57% of all genes. This number is remarkably similar to the 5.43% figure in Table 3 (G8) regarding the proportion of up/downstream variants, genome-wide, that are in SFARI-listed genes, validating our choice of using these variants as controls. If we errored and some proportion of the up/downstream variants in our subjects indeed are disease/ASD-related, that would skew our findings toward the null hypothesis. Thus, we may have mildly underestimated the importance of de novo (and silent) variants in this study. All variant types we queried, both de novo and inherited, were found to be statistically more likely to be in SFARI-listed genes than are control (upstream/downstream) variants among our subjects (Table 3, row 14, pink background).

Our data demonstrate that inherited silent variants are highly more likely than control (inherited up/downstream) variants to be in SFARI-listed genes (*p* < 0.0001, Table 3, E14). Unexpectedly, these inherited silent variants are also highly more likely than inherited missense variants to be in SFARI-listed genes (*p* < 0.0001, D12, orange background). Additionally, despite small numbers, de novo silent variants are increased relative to control variants and have similar odds ratios as those for de novo missense variants (compare cells B14 and C14).

## 4. Discussion

### 4.1. Phenotypes in ASD

Overall, the current and previous study [14] cohorts are quite clinically similar in terms of the proportion that is female (20% versus 22%), nonverbal (29% vs. 26%), epileptic (27% vs. 30%), and post developmental regression (57% vs. 54%), respectively. As explained in [14], these parameters are rather typical for people with autism seen by tertiary care specialists. Tics are less common in the current cohort (9% vs. 26%, *p* = 0.013), although this is difficult to assess from chart review as parents often confuse tics with other conditions, such as repetitive autistic behavior, and may reflect practice methodology differences between the two physicians. However, the current cohort demonstrates an overall significantly higher severity of intellectual disability (ID), in that at least moderate ID is present in 85/95 (89% with 5 subjects not recorded) versus in 25/50 (50%) of the subjects we reported previously [14] (*p* < 0.0001). Although the numbers are small, individuals who were less affected in terms of intellectual disability, nonverbal status, epilepsy, or past developmental regression appear to have fewer important (PDVs) DNVs identified. Thus, the results of our study should be interpreted as applying to a cohort of predominantly children and young adults with autism on the more severe end of the spectrum that are referred to a tertiary care specialist. Lower genomic yields are possible, and likely expected, in individuals less clinically affected.

The polygenic nature of ASD [24] and the large number of involved genes preclude genotype–phenotype correlations in a study of this size. To address these correlations, phenotypic and genotypic information at least as detailed as tabulated in the present study in very large ASD cohorts will need to be reviewed, likely through a meta-analysis. The information presented in Table S1 and Table 1 can be used in such analyses. In addition, this information might be useful when additional cases are identified with DNVs in genes corresponding to the 24 Novel (no cases reported) and 19 Very rare (one to nine cases reported) disorders (Table 2) briefly characterized herein.

### 4.2. Genotypes in ASD

Physicians are aware of monogenic disorders, in which one to two variants in a single gene are predominately causal for disease (e.g., cystic fibrosis, sickle cell), and highly polygenic disorders, in which multiple common variants each contribute only a small degree of the genetic susceptibility (e.g., asthma, diabetes). Of course, in real life, there are numerous shades of grey between these models, and that is where ASD apparently oftentimes lies [24]. An inherited genetic variant in an unaffected or minimally-affected parent is unlikely to be a substantial risk factor for severe disease in their child (disease-causal or major risk factor), unless bi-allelic/recessive, but certainly could be a less substantial (intermediate or minor) risk factor, or unrelated. On the other hand, a DNV in that setting could be a risk factor with any degree of disease association, from disease-causal to unrelated.

Another approach would be to use a control group matched to important characteristics of the individuals with ASD in unaffected individuals without any first-degree relatives with a neurodevelopmental disorder. However, given the wide range of comorbidities seen in people with ASD, matching controls to many of the important comorbidities and other characteristics would mostly likely be incomplete if not impossible. Using the individuals as their own controls provides a tight matching to these characteristics.

Our best option was to look at SFARI status for each gene with an identified variant in the subjects. Listing genes with high degrees of certainty to be ASD-related, the SFARI database only lists a relatively small fraction of genes related to ASD based on a detailed literature search (Table 2, compare the fourth column to the fifth column). However, the latter analysis is extremely labor-intensive and thus not possible to use to score the thousands of genes in which inherited variants were identified. However, the SFARI status of the gene could be, and was, automated for every variant found. If missense variants throughout all genes are indeed part of the background genetic predisposition toward ASD (e.g., minor risk factors), the genes in which these variants are found should be weighed toward having more ASD-related genes, relative to controls. This is indeed what we are reporting. The comparison between de novo PDVs versus control (up/downstream) variants (Table 3, cell B14) is the primary test of the main point of this paper, and reflected in its title, that de novo variants are “predominant” in ASD. This is one of only two pre-analysis (conceived prior to data analyses) comparisons in this study. These data are highly statistically significant (*p* < 0.0001) and remain so following Bonferroni correction.

The number of variants and genes comprising this background/minor inherited risk among our cohort is large. For example, we identified an average of 157 inherited missense variants per subject, 14 (9%) of which are in SFARI-listed genes (Table 3, column D). Given the odds ratio of 1.7 versus controls (Table 3, cell D14), this means that missense variants in the cohort are 70% more likely to be in a SFARI gene relative to controls (50% increased odds if using the lower figure in the 95% confidence interval). The comparison of inherited missense to inherited control variants is the second and final pre-analysis comparison in this study. This comparison is highly statistically significant (*p*< 0.0001) and remains so following Bonferroni correction. For missense DNVs, we identified an average of 0.43 variants per subject, 0.11 (27%) of which are in SFARI-listed genes, with an odds ratio of 5.8 (Table 3, column B). This is an almost six-fold increased likelihood, suggesting that the majority (6:1) of the DNV-PDVs we present in the current tables are predicted to be ASD-related in those subjects. Again, and as per [24], we assert that DNVs in ASD can be benign, minor risk factors, major risk factors, or disease-causal, but that when strict criteria are imposed (like those we use to define PDVs), most of those identified are disease-related to various degrees. As a comparison, 1.5 DNVs per subject are present in our current ASD cohort, versus 0.2–0.3 per person in unaffected people (discussed in [14]).

### 4.3. Silent Variants in Autism

Silent, also known as synonymous, variants occur when a mutation in the third nucleotide of a codon does not change the amino acid code. Silent variants can affect the gene expression of proteins through various mechanisms, including changes to mRNA binding, microRNA, RNA splicing, and codon efficacy, among others. However, these effects are generally difficult to measure, and silent variants are often overlooked, particularly in clinical medicine. In our previous study, we used silent variants as controls (sic), and intended to do the same in the current study until we analyzed the data. However, as per the Results section and Table 3, our data reveal that silent variants, both inherited and de novo, are strongly associated with ASD in our cohort. Among inherited variants, silent variants are more likely to be in SFARI genes than are control (up/downstream) variants (*p* < 0.0001, OR 1.7, 95% C.I. 1.5–1.9). Inherited silent variants are also more likely to be in SFARI genes than are inherited missense variants (*p* < 0.0001, 1.3, 1.2–1.5). Among de novo variants, silent and missense variants have similar odds ratios, relative to controls, for being in SFARI genes (OR, 95% C.I.: 5.8, 2.9–11 versus 4.6, 1.5–14). The three comparisons in the Abstract section regarding silent variants are all post-analysis; thus, these should be considered to be putative despite remaining highly significant following Bonferroni corrections.

Takata et al., 2016 [25] “found that near-splice site de novo synonymous mutations are almost twice as frequent in ASD than controls” (*p* = 0.0003, OR 1.96), identifying “101 mutations in 1043 ASD cases and 37 mutations in 731 controls”. The estimated contributions of de novo silent variants were “comparable to that of” de novo loss-of-function variants (1.3%), “and much higher than that of” de novo missense variants (0.1%). Per Jaganathan et al., 2019 [26], “(d)e novo mutations that are predicted to disrupt splicing are enriched 1.51-fold in intellectual disability (*p* = 0.000416) and 1.30-fold in autism spectrum disorder (*p* = 0.0203) compared to healthy controls”. Rhine et al., 2022 [27] wrote that “(e)xonic splicing mutants were enriched in probands relative to unaffected siblings—especially synonymous variants (7.5% vs. 3.5%, respectively)”. An increase in silent postzygotic mosaic mutations was published in one study [28]. In addition, silent DNVs were published as being causal for ASD in at least three case reports [29,30,31].

The literature and the current data suggest that silent variants are important in ASD pathogenesis, perhaps with a higher disease association than missense variants, with odds ratios relative to controls varying from 1.3 to 2.0 (1.7 in the present study; the higher end of 2.0 was for near-splice site variants). Thus, silent DNVs perhaps should not be dismissed categorically when evaluating the DNA sequence of someone with ASD. Based on this information, 17 silent DNVs were re-scored as PNVs, which increased the number of our 100 subjects that have at least one DNV-PNV by 8, from 47 to 55.

### 4.4. ACMGG Criteria, near Misses, and Low Laboratory Yield

The main limitation of this study is the difficulty of determining if a variant alters protein function or is disease-related, which is also the main limitation in clinical genetic testing. In our determination of a DNV as a potential PDV, we aimed for a higher specificity (identifying less false positives) such that variants so designated are highly likely to be disease-related. As in our previous work [14], we used American College of Medical Genetics and Genomics (ACMGG) standards as much as possible and clearly stated any deviations. In particular, ACMGG guidelines are not designed for research on novel disorders, but for clinical analysis regarding known disorders. Indeed, the vast majority of the listed DNV-PDVs we report (Table 1 and Table 2) at least meet ACMGG criteria for Likely Pathogenic (based on PS2/DNV and PM2/not present in control individuals), with the only caveat that we applied PM2 for prevalence <0.00001 (<1 in 10,000 alleles). The ACMGG guidelines were published in 2015 when control sequences were limited, while current databases constitute about one million individuals. We added the requirement for at least moderate evolutionary conservation, which is beyond the ACMGG guidelines. Furthermore, the ACMGG guidelines are designed for known disorders, while we also wished to identify variants in very rare and novel disorders. Since we cannot rely on phenotypic matches for these disorder types, in order to increase specificity, we only scored as PDVs variants in genes published to be related to ASD, either directly or one-degree indirectly, as per the Subjects and Methods section. We believe that our modifications preserve the spirit and intent of the ACMGG guidelines, allow their translation into novel disorders, and require evolutionary conservation to increase specificity.

In the process of strengthening specificity, sensitivity is compromised. There are many “near-misses”, or DNVs that might be disease-related, but were not labeled as such due to failure to meet a single criterion. Two such examples include the frameshift variants in subjects #24 (CD101) and 38 (ZNF300), excluded for no known connection of the genes to ASD even though both are in pathways (immune, gene expression) known to be important in ASD pathogenesis (B1 genes). In particular, new pathways related to ASD will be missed by our, or any (e.g., ACMGG), methodology that requires a published connection to ASD for each gene in variant classification. A variant in another zinc finger gene (ZNF516) in subject #31 was excluded on grounds of conservation only. A variant in TWF2 in subject #19 was excluded for a combined prevalence figure just barely >1/10,000. A review of Table 1 reveals many other similar near-misses.

Based alone on a laboratory report of indeterminate or better, the yield for at least one DNV is only 15/100 (15%) in our cohort. However, this figure jumps to 55/100 (55%) for having at least one DNV-PDV following our methodology, providing additional DNVs highly likely to be disease-related in 40/100 (40%) additional subjects. There are many reasons for this discrepancy, but mostly the present methodology did not exclude the following: (1) very rare and, especially, novel disorders, which are beyond the preview of clinical laboratories, (2) very rare variants that are nonetheless still listed over zero prevalence in the over-one-million-person gnomAD control database, and (3) silent DNVs. Our methodology requires expertise in genomics and the pathophysiology of ASD; thus, it is not suitable for widespread adoption, although it is the clinical practice for all ASD patients seen by the first author.

### 4.5. Mechanistic Pathways and Clinical Utility

There are a variety of mechanistic pathways known to be involved in ASD, many of which are shown in Table 2 corresponding to the known functions of genes in which we identified a DNV-PDV. Most of these pathways are either neuron/brain-specific (synapse-related, neurogenesis) or ubiquitous to all cell types but of particular importance to neurons/the brain (ion transport, energy metabolism, immune system, ubiquitin-related protein turnover, cytoskeleton, cell–cell interactions, and cell danger response). The remaining pathways, gene expression and cell signaling, are highly complex as tissue specificity varies from case to case. Together, these pathways are highly fundamental to biology, in general. Considering the issue of multiple factors leading toward ASD from another direction, despite over 5% of all genes being listed on SFARI, that database likely only includes a small fraction of genes in which variants can predispose toward autism. Thus, a very sizable proportion of all genes is likely involved in ASD pathogenesis. How do so many genes in a variety of pathways fundamental to life predispose toward one entity—ASD? A parsimonious hypothesis is that social communication and executive functioning (e.g., ADHD, which is extremely common in ASD [32]) are highly vulnerable pathways that are oftentimes the main sequalae of generalized cellular insults (e.g., hypoxia, ethanol) and, thus, are oftentimes the main sequalae of a DNV in a very large number of genes in which the variant severely compromises general cellular homeostasis. Phenotypic targeting (e.g., the development of ASD versus intellectual disability, epilepsy, schizophrenia, etc.) may be in large part due to inherited genetic modifying variants and/or environmental factors. Future studies are needed to continue exploring the pathways that contribute to ASD to find additional actionable clinical targets.

Of particular clinical importance is that four of the pathways shown (ion transport, energy metabolism, synapse-related, and immune system) are at least partially treatable. In the clinical practice of the first and last authors, identifying variants, both de novo and inherited, in these pathways among our ASD patients frequently leads to treatment options with anecdotal clinical improvements.

### 4.6. Limitations

As explained above, the main limitation of this study is the difficulty in variant classification in terms of disease relationships, including the difficulty of determining if a gene is ASD-related. Our strict criteria likely led to an under-ascertainment of DNVs associated with ASD. Our cohort is small in terms of a sequencing study in ASD, but large for a study that correlates phenotype, genotype, and mechanisms. Hopefully, future studies will include more subjects as well as this information. Modifications to the ACMGG standards were made to better adapt to the analysis of new genes, and, in doing so, this study varies from that in many other reports. Computational-modeling evidence is provided for missense variants, but not for silent variants, and functional validation of variant pathogenicity is absent for both variant classes. Thus, conclusions are premature, especially regarding silent variants. Finally, our cohort represents individuals on the more severe end of the broad ASD spectrum and is likely not applicable to those with lesser degrees of clinical severity.

### 4.7. Potential Implications for the Increasing Prevalence of ASD

At the time of this publication, the autism community is bitterly divided among those that believe that ASD is genetic and not increasing in frequency (e.g., better recognition, altered diagnostic practices) and those that believe that the frequency is increasing dramatically, which can only be due to environmental toxicity. DNVs, which are genetic yet not inherited, occur in both monozygotic twins but only one dizygotic twin, and, thus, would be determined to be genetic in twin studies. DNVs generally occur in spermatogenesis (small variants) or oogenesis (CNVs), while a relatively small proportion of DNVs are postzygotic in early embryology. Most of the DNVs found in autism are small variants, usually single-nucleotide, thus likely occurring in spermatogenesis, possibly years or even decades prior to conception. DNVs are not new; they have always occurred. Indeed, they are the drivers of evolution as well as new genetic disorders. However, and herein the authors speculate, the rapidly increasing incidence of ASD might be due to an increasing rate of DNVs caused by mutagenesis secondary to multiple and dramatic environmental changes occurring in recent decades. In particular, heavy metals, chemicals such as dibenzodioxins and alkylating agents, and multiple metabolites from bacteria and fungi are known to be mutagenic.

In addition, insufficient folate during gestation or gametogenesis can result in DNVs (mutations) [33,34,35]. Insufficient folate during early gestation can cause postzygotic DNVs, while prezygotic maternally derived DNVs occur in the maternal grandmother during gestation of the mother. Additionally, prezygotic paternally derived DNVs can continue to occur during spermatogenesis, which commences in adolescence and continues throughout life. One of the unique characteristics of ASD is the relationship between paternal age and increasing ASD risk. Advanced paternal age provides more time for DNVs to occur. Toxicant exposure and poor folate intake throughout life could certainly result in a cumulative mutation load resulting in poorer sperm quality in age. Interestingly, folate is protective for environmental toxicants, so suboptimal folate intake itself may not cause DNVs but could increase the risk of toxicants causing DNVs. On the other hand, excessive folic acid may increase the DNV rate [36].

The authors assert that future studies are extremely important to answer the questions posed by our work. Are DNVs in humans increasing over time? Are they more numerous in people with ASD, or are those people simply unlucky as to where the DNVs occurred? What environmental factors are driving any increase in DNVs? Finally, perhaps a question that all aspects of the ASD community can agree on: What environmental epigenetic factors are contributing to ASD pathophysiology, whether the targeted genetic variants are de novo or inherited, and regardless of whether DNVs are (or ASD is) truly increasing in prevalence over time?

## 5. Conclusions

DNVs, including missense and silent, are likely related to disease pathogenesis in about one half of individuals with moderate-to-severe forms of ASD, likely as significant factors in disease pathogenesis. This statement is caveated by the lack of an unaffected control group. Numerous inherited variants, including missense and (provisionally) silent, are ASD-associated, likely each as minor factors in disease pathogenesis. DNVs can explain how a predominately genetic disorder could rapidly increase in true incidence and themselves can oftentimes suggest therapeutic options. However, knowledge in this area is still preliminary, and future studies are desperately needed.

## Figures and Tables

**Table 1:** All coding de novo variants identified in our 100 subjects with ASD.

Subject	De Novo Small Variants Identified	Variant	Gene ASD	SFARI	Allelic Prevalance	Allelic Prevalance	Conservation	Conservation	Conservation
Number		Type	Relation ^1^		gnomAD, #1 ^2^	gnomAD, #2 ^2^	PhyloP	PhastCons	UCSC GB ^3^
1	SMC4_c.1472C>T p.Ser491Leu chr3:160,417,757	Missense	B0	not	0	0.0000048	0.07,P	0.987,D	Low in mammals
	SLC12A1_c.1473G>T p.Gly491= chr15:48,246,929	Silent	B0	not	0	0	NA	NA	
	GRIK5_c.693G>A p.Ser231= chr19:42,056,973	Silent	A2	2	0.000026	0.000014	NA	NA	
2	MYLK_c.3902G>A p.Arg1301His chr3:123,664,188	Missense	A3	3	0.0000066	0.00004	0.935,D	0.52,B	High in mammals
	DKK2_c.243T>C p.Asp81= chr4:106,925,929	Silent	A3	not	0	0	NA	NA	
3	ANK2_c.1243G>A p.Glu415Lys chr4:113,258,104	Missense	A1	1	0	0	1.045,D	1.0,D	
	MRPL27_c.65C>T p.Pro22Leu chr17:50,370,562	Missense	A3	not	0.000085	0.000035	−0.836,B	0.0,B	
	MYO5B_c.93C>T p.Tyr31= chr18:50,055,313	Silent	B0	not	0	0	NA	NA	
4	BFSP1_c.1100C>G p.Pro367Arg chr20:17,494,972	Missense	B1	not	0	0	0.953,D	0.92,D	
5	UNKL_c.1683G>C p.Ser561= chr16:1,367,761	Silent	B0	not	0	0	NA	NA	
6									
7	CO1_m.6324G>A, p.Ala141Thr; heteroplasmy: subject 20%, mother 4%	mtDNA ^4^	A1	1	0.00011	0.00003544	3.56481D		
8	IQGAP2_c.1518C>T p.Leu506= chr5:76,611,180	Silent	B0	not	0.000024	N/A	N/A	N/A	
9	MOGAT3_c.636_646, 650_652del14nt frameshift chr7:101,198,206	Frameshift	B3	not	0	0	NA	NA	
10	ASXL1_c.3437C>A p.Ser1146Ter chr20:32,436,149	Nonsense	B0	not	0	0	N/A	N/A	
	APLP1_c.685C>T p.Arg229Trp chr19:35,871,871	Missense	A3	not	0.000039	0.000092	0.935,D	0.935,D	
	COL4A4_c.4314C>T p.Asp1438= chr2:227,012,200	Silent	B3	not	0.000046	0.000034	N/A	N/A	
11	SLC6A1_c.28G>A p.Asp10Asn chr3:11,017,239	Missense	A1	1	0.0000031	0	0.953,D	0.968,D	
	DSCAM_c.182C>A p.Ala61Asp chr21:40,708,633	Missense	A1	1	0	0	0.998,D	0.998,D	
	FAM151A_c.1417C>G p.His473Asp chr1:54,609,609 (possible paternal gonadal mosaicism)	Missense	B2	not	0.000047	0	0.138,P	0.961,D	High in mammals
	MTCL1_c.3962C>T p.Pro1321Leu chr18:8,819,108	Missense	B0	not	0.0000081	0	0.935,D	0.142,B	Low in mammals
	NPAS3_c.2196C>T p.Gly732= chr14:33,800,503	Silent	A3	not	0.0000039	0	N/A	N/A	
	TBPL1_c.390C>T p.Tyr130= chr6:133,984,580	Silent	B1	not	0.000011	0	N/A	N/A	
12	SETDB2_c.457A>G p.Met141Val chr13:49,476,591	Missense	A2	2	0	0.0000064	1.199,D	0.548,B	Low in mammals
13	EFR3B_c.2379T>C p.Thr793= chr2:25,154,265	Silent	A3	not	0.00002	0.0000077	NA	NA	
	monosomy X 35% mosaic	CNV-del	B2	A1	common				
14	KIDINS220_c.4513A>T p.Thr1505Ser chr2:8,731,523	Missense	A3	not	0	0	1.061,D	1.0,D	
	GLT6D1_c.757G>A p.Asp253Asn chr9:135,624,171	Missense	B3	not	0	0	0.892,D	0.003,B	High in vertebrates
	RIMS1_c.1575G>A p.Lys525= chr6:72,183,046	Silent	A1	not	0	0	N/A	N/A	
15	SCNN1A_c.1093G>A p.Gly365Ser chr12:6,355,322	Missense	B2	not	0	0	0.852,D	0.984,D	
16	MAST3 c.1963T>C, p.Phe684Leu, chr19:18,137,316	Missense	A2	not	0	0	0.922,D	0.989D	
	SH3RF1_c.2583A>G p.Lys861= chr4:169,096,603	Silent	A3	3	0	0	NA	NA	
17	SHANK3_c.3658dupG p.Ala1289GlyfsTer82 chr22:50,721,504	Frameshift	A1	1	0	0.00002	N/A	N/A	
18	RMND5B_c.238A>G p.Lys80Glu chr5:178,142,681	Missense	A3	not	0	0	1.194,D	0.976,D	
	WDR1_c.718A>G p.Ile240Val chr4:10,087,940	AR missen	B0	not	0	0	0.146,P	0.922,D	High in amniotes
19	SERPINB3_c.380A>G p.Tyr127Cys chr18:63,658,602;	Missense	B2	not	0		0.025,P	0.004,B	
	TWF2_c.563G>A p.Arg188Gln chr3:52,230,916	Missense	B0	not	0.000099	0.00014	0.852,D	0.925,D	
	Xp11.4p11.4x4(37,818,872-37,842,030) 23.16kb Duplication 79% of DYNLT3	CNV-dup	A3	not	0		NA	NA	
	22q11.21q11.21x1(18,878,000-19,041,500) 163.50kb Deletion of 8 genes, incl PRODH	CNV-del	A2	2	0.0039		NA	NA	
20									
21	PDCD5_c.240T>C p.Tyr80= chr19:32,585,889	Silent	B1	not	0	0.0000055	N/A	N/A	
	19q13.33q13.33x3(50,409,797-50,476,028) 66.23kb Duplication of 5 genes, incl 63% of POLD1	CNV-dup	B0	not	0				
22	ABCB6_c.1900C>T p.Arg634Cys chr2:219,212,455	Missense	A3	not	0.000026	0.000032	1.044,D	0.997,D	
23									
24	ARHGEF2_c.763A>T p.Lys255Ter chr1:155,963,145	Nonsense	A3	3	0		1.042,D	0.964,D	
	CD101_c.1078_1079delTT p.Phe360LeufsTer12 chr1:117,013,640	Frameshift	B1	not	0		N/A	N/A	
25	COL6A3_c.3424G>A p.Asp1142Asn chr2:237,374,667	Missense	B0	not	0.00002	0.000024	0.935,D	0.002,B	High in mammals
	KLHDC4_c.272C>G p.Thr91Ser chr16:87,755,291	Missense	B1	not	0		1.048,D	0.064,B	High in vertebrates
	JPH3_c.1791C>T p.Gly597= chr16:87,690,151	Silent	A3	not	0	0.0000049	N/A	N/A	
	COL18A1_c.1551A>G p.Gly517= chr21:45,480,798	Silent	B3	not	0	0	N/A	N/A	
26									
27	PLEKHH2_c.1636C>T p.Arg546Cys chr2:43,700,594	Missense	B0	not	0.000013	N/A	0.935,D	1.0,D	
	GHRHR_c.1241C>T p.Ser414Leu chr7:30,979,213	AR missen	B1	not	0.000046	0.000031	0.054,P	0.008,B	
28	TBC1D3I_c.1161G>A p.Arg448= chr17:36,254,443 (possible mosaic)	Silent	B2	not	0.000092	0	N/A	N/A	
29	SLC4A11_c.2542_2550dupGCCATGATC p.Ala848_Ile850dup chr20:3,228,266	AR insert	B0	not	6.20 × 10^−7^	0	N/A	N/A	
30	MGA_c.5107G>A p.Ala1752Thr chr15:41,743,067	Missense	B3	not	0		0.225,P	0.999,D	Low in mammals
	TYK2_c.1438C>T p.Pro480Ser chr19:10,362,587	AR missen	A3	not	0		0.953,D	0.966,D	
	MT-CYB m.15021T>C, p.Ile92Thr; heteroplasmy: subject 15%, mother 0%	mtDNA	A1	1	0	0.000018	3.74109		
31	AQP2_c.315T>G p.His105Gln chr12:49,951,145 (possible mosaic)	Missense	B0	not	0	0	−0.224,B	0.956,D	High in mammals
	ZNF516_c.2998C>T p.Arg1000Cys chr18:76,379,116	Missense	B0	not	0.000046	0.00005	0.108,P	0.335,B	
32	KDM5B_c.1876G>A p.Val626Met chr1:202,749,085 de novo ^5^	Missense	A1	1	0	N/A	0.876,D	0.855,B	High in vertebrates
	* KDM5B_c.1466T>C p.Val489Ala chr1:202,755,343 paternal, phase unknown ^5^*	*Missense*			*0.000013*	*0.000016*	*1.082,D*	*1.0,D*	
33	APBB1_c.1217delA p.Asn406ThrfsTer26 chr11:6,402,612	Frameshift	A2	2	0		N/A	N/A	
	TMEFF1_c.122A>C p.Asn41Thr chr9:100,473,666 possible somatic mosaic	Missense	A3	not	0		0.988,D	0.996,D	
	GARRE1_c.2697G>A p.Leu899= chr19:34,349,025	Silent	B3	not	0		N/A	N/A	
34	ASPM_c.7662_7663delCA p.His2554GlnfsTer14	Frameshift	A2	2	0.0000031		N/A	N/A	
	CBARP_c.1154+1G>A chr19:1,231,100 Splicing-Donor ^6^	Splice	B0	not	0	0	0.838,D	1.0,D	
35	EFCAB13_c.2448T>C p.Asp816= chr17:47,414,873	Silent	B1	not	0		N/A	N/A	
36									
37	HNRNPDL_c.644G>C p.Gly215Ala chr4:82,428,148	Missense	B0	not	0	0	0.935,D	1.0,D	
38	FLNB_c.4361C>T p.Pro1454Leu chr3:58,130,879	Missense	B2	not	0.0000066	0.000014	0.935,D	0.509,B	High in vertebrates
	ZNF300_c.660dupA p.Ser221IlefsTer3 chr5:150,896,578	Frameshift	B1	not	0		N/A	N/A	
39	9p22.3p22.3x4(15,405,655-15,517,446) 111.79kb Duplication, heterozygous	CNV-dup	A3	A3	0		N/A	N/A	
	* 3 genes, 2 coding: SNAPC3 (an A3 gene); PSIP1 (an A3 gene)*								
40	10q21.3q22.2x1(65,164,362-74,517,047) 9.35Mb Deletion; multiple genes	CNV-del	A2	2	0.0004	Pathogenic			
	* 3 SFARI (ADK (51% of coding region), CTNNA3 (100%), AGAP5 (100%)-all SFARI-2*								
41	CNDP1_c.216A>C p.Gln72His chr18:74,559,385	Missense	B0	not	0		−0.128,P	0.912,D	Low in mammals
	CLCN4_c.984C>G p.Tyr328Ter chrX:10,208,185	Nonsense	A2	2	0	0	0.064,P	1.0,D	
	SLC4A4_c.1859G>T p.Gly620Val chr4:71,472,926	AR missen	A3	not	0	N/A	0.953,D	0.996,D	
42	CD101_c.2716A>G p.Met906Val chr1:117,025,796	Missense	B1	not	0	0	−0.275,B	0.706,B	
	MUC12_c.6535G>T p.Gly2179Cys chr7:100,997,098 (possible mosaic)	Missense	A2	2	0	0	0.313,P	0.004,B	
43									
44	INS_c.16C>A p.Arg6Ser chr11:2,160,956	Missense	B2	not	0	0	0.143,P	0.104,B	
	16p11.2p11.2x1(29,520,000-30,226,500) 706.50kb Exonic Deletion	CNV-del	A1	1	0.001	Pathogenic			
	30 genes, 5 SFARI: CORO1A (SFARI-1); SEZ6L2, KCTD13, TAOK2, MAPK2 (all SFARI-2)								
45	SMCHD1_c.4220C>G p.Pro1407Arg chr18:2,751,332	Missense	B2	not	0	0	0.892,D	0.999,D	
	VPS13B_c.6490A>C p.Asn2164His chr8:99,699,893	AR missen	A1	1	0	0	1.199,D	0.985,D	
46	CPVL_c.1254T>C p.Val418= chr7:29,030,643	Silent	A3	not	0.0000066	0	N/A	N/A	
47									
48	KBTBD13_c.1312A>G p.Thr438Ala chr15:65,078,127	Missense	B0	not	0.000012	0	1.199,D	0.492,B	High in vertebrates
49	TTN_c.6163G>A p.Glu2055Lys chr2:178,775,701	Missense	A2	2	0.000033	0.000058	0.852,D	0.58,B	High in mammals
50	TTN_c.83875A>G p.Ile27959Val chr2:178,562,257	Missense	A2	2	0	0	1.061,D	0.996,D	
	MT-CYB m.15586T>C p.Ile280=; heteroplasmy: subject 66%, mother 33%	mtDNA	A1	1	0.00048		N/A	N/A	
51	NNAT_c.230A>G p.Gln77Arg chr20:37,522,743	Missense	B0	not	0.000027	0	1.197,D	1.0,D	
	HMCN2_c.4981C>A p.His1662Asn chr9:130,354,882	Missense	B2	not	0	0	0.852,D	0.977,D	
	TNFRSF25_c.455G>A p.Arg152Gln chr1:6,464,560	Missense	B1	not	0.0000074	0	−0.371,B	0.002,B	
52	MLXIPL_c.2336T>C p.Phe779Ser chr7:73,594,378	Missense	A3	not	0	0.000005	1.18,D	0.995,D	
	SLC4A5_c.2861C>T p.Pro954Leu chr2:74,227,865	Missense	A3	not	0.0000066	0.0000099	1.048,D	0.975,D	
53									
54	ARHGAP8_c.611A>G p.Asn204Ser chr22:44,845,283	Missense	B0	not	0		1.199,D	0.706,B	
	GDI2_c.571C>A p.Leu191Ile chr10:5,785,868	Missense	B0	not	0		1.048,D	1.0,D	
	MT-CYB m.15209T>C, p.Tyr155His; heteroplasmy: subject 34%, mother 17%	mtDNA	A1	1	0.00014		5.585,D		
55									
56									
57									
58									
59									
60	ARHGEF18_c.2325C>A p.Asn775Lys chr19:7,458,655	AR missen	B2	not	0	0	N/A	N/A	
61	KCNJ6_c.353G>A p.Arg118Gln chr21:37,714,804	Missense	A3	not	0.0000066		0.935,D	0.983,D	
	HECW1_c.3340+1G>T chr7:43,492,181; spliceRF 0.928, spliceADA 0.99999	Splice	A3	not	0		0.953,D	0.998,D	
62	CXCR1_c.448C>T p.Arg150Cys chr2:218,164,764	Missense	B0	not	0.00006	0	−0.344,B	0.177,B	High in vertebrates
	USP20_c.149A>G p.Tyr50Cys chr9:129,858,063	Missense	A3	not	0	0	1.199,D	0.971,D	
63	ZNF865_c.1718C>T p.Thr573Met chr19:55,615,336	Missense	B0	not	0	0	0.867,D	0.964,D	
	CNOT11_c.510C>G p.Leu170= chr2:101,253,474	Silent	B0	not	0.000013	0	N/A	N/A	
64	ERF_c.205G>A p.Val69Ile chr19:42,250,383	Missense	B0	not	0.000019	0	0.935,D	0.967,D	
65									
66	TRPV4_c.97C>T p.Leu33Phe chr12:109,814,700	Missense	A3	not	0	0.0000032	0.885,D	0.997,D	
	GPS1_c.328G>A p.Asp110Asn chr17:82,054,529	Missense	A3	not	just <0.0001	0.000022	0.953,D	0.338,B	High in vertebrates
67									
68	OXLD1_c.281G>A p.Gly94Asp chr17:81,665,364	Missense	B2	not	0.000085	just <0.0001	0.836,D	0.998,D	
	SETD1A_c.663C>T p.Ser221= chr16:30,964,117	Silent	A1	1	0.0000066		N/A	N/A	
69									
70	NR1I2_c.250T>A p.Cys84Ser chr3:119,810,113	Missense	B2	not	0		0.964,D	0.996,D	
71	LRTM1_c.143T>C p.Leu48Ser chr3:54,925,080	Missense	B0	not	0		1.199,D	0.729,B	High in vertebrates
	RACK1_c.824T>C p.Ile275Thr chr5:181,237,673	Missense	B0	not	0		1.199,D	0.998,D	
	AHNAK_c.16293C>T p.Gly5431= chr11:62,518,124	Silent	A2	2	0.000085	0.00011	N/A	N/A	
	MT-RNR2 m.2672A>G, rRNA; heteroplasmy: subject 35%, mother 11%	mtDNA	A1	1	0.00021	0.000018			
72	DMXL1_c.1083C>T p.Ala361= chr5:119,121,120	Silent	A3	not	0		N/A	N/A	
	16p11.2p11.2x3(28,744,500-29,065,000) 320.50kb Duplication	CNV-dup	not	not	0.0005		N/A	N/A	
	14 genes, 10 coding, none SFARI								
73	8p23.3p23.1x1(163,500-7,383,000) 7.22Mb Deletion; many genes	CNV-del	A2	2	0		N/A	N/A	
	* 5 SFARI genes: CLNB, ARHGEF10 MCPH1, DLGAP2, CSMD1-all SFARI-2*								
	1q21.2q21.2x3(150,442,000-150,519,000) 77.00kb Duplication	CNV-dup	A3	not	0		N/A	N/A	
	5 genes, 3 coding, none SFARI; RPRD2 is an A3 gene								
74	MAP4K1_c.994G>C p.Ala332Pro chr19:38,609,608	Missense	A3	3	0	N/A	0.847,D	0.997,D	
75	MT-ND5 m.13119C>T, p.Ile261=; heteroplasmy: subject 31%, mother not done	mtDNA	A1	1	0.00%		N/A	N/A	
76									
77	SLC4A8_c.3160A>T p.Asn1054Tyr chr12:51,504,107	Missense	A3	not	0	N/A	1.088,D	0.973,D	
	ERAP1_c.1817G>A p.Gly606Asp chr5:96,785,914	Missense	B0	not	0	N/A	0.932,D	0.999,D	
	GK2_c.795_796insAAGGT p.Gly266LysfsTer38 chr4:79,407,405	Frameshift	B3	not	0	N/A	N/A	N/A	
	PTPRS_c.1227C>T p.Gly422= chr19:5,244,205	Silent	A3	not	0.00002	0.000015	N/A	N/A	
	TOX_c.612A>G p.Ser204= chr8:58,851,605	Silent	B0	not	0		N/A	N/A	
78	RFXANK_c.216T>G p.Thr72= chr19:19,196,991 [also, AR]	AR silent	B0	not	0	0	N/A	N/A	
79	STKLD1_c.575C>T p.Ala192Val chr9:133,390,788	Missense	B3	not	0.00004	0.000056	0.848,D	0.381,B	High in mammals
80									
81	COLEC12_c.674G>A p.Arg225Gln chr18:346,948	Missense	B0	not	0.000079	0.00023	0.077,P	0.997,D	High in mammals
	LINC02203_c.195C>T p.Asn82= chr15:21,652,129	Silent	B3	not	0.0000083		N/A	N/A	
82									
83	STAB1_c.5470A>G p.Ile1824Val chr3:52,520,261	Missense	B0	not	0.0000066	0.000053	−0.117,P	0.941,D	High in mammals
84	DCAF4L2_c.1161G>T p.Glu387Asp chr8:87,872,811	Missense	B0	not	0	N/A	−0.244,B	0.2,B	Poor alignment
	KCNA10_c.922G>A p.Asp308Asn chr1:110,517,866	Missense	B1	not	0	N/A	0.935,D	0.897,B	High in vertebrates
	GLB1L3_c.1035C>A p.Thr345= chr11:134,309,699	Silent	B2	not	0.000046	N/A	N/A	N/A	
85	OR6P1_c.904A>G p.Arg302Gly chr1:158,562,701	Missense	B2	not	0	N/A	1.011,D	0.997,D	
	MEIOB_c.1072_1073delAT p.Met358ValfsTer12 chr16:1,839,399	Frameshift	B2	not	0.000033	0.000025	N/A	N/A	
86									
87	FMN1_c.3941A>G p.His1314Arg chr15:32,804,320	Missense	A3	not	0	0.000002834 (4/1411316)	1.061,D	0.986,D	
88									
89	SHROOM2_c.3662C>A p.Pro1221Gln chrX:9,937,208	X-miss	A3	not	0	0	0.935,D	0.932,D	
	PLOD3_c.1585G>A p.Asp529Asn chr7:101,210,360	AR missen	B1	not	0	0.00001	0.859,D	0.862,B	High in vertebrates
90									
91	PTGFR_c.923G>A p.Arg308Gln chr1:78,536,530	Missense	B0	not	0.0000087	0	0.953,D	0.997,D	
	MT-CO3_m.9210A>T, p.Thr2Ser; heteroplasmy: subject 44%, mother 34%	mtDNA	A1	1	0	0	−0.833,B		
	MT-ATP6_m.8854G>A, p.Ala110Thr; heteroplasmy: subject 20%, mother 0%	mtDNA	A1	1	0.0011	0.00016	−0.031,P		
92	DCHS1_c.8209delG p.Ala2737LeufsTer24 chr11:6,623,466 ^7^	AR Frames	A3	not	0	0	N/A	N/A	
93	EHBP1L1_c.3755G>A p.Arg1252Lys chr11:65,585,413	Missense	B1	not	0	0	0.053,P	0.024,B	
	HDLBP_c.2950C>T p.His984Tyr chr2:241,235,549	Missense	A1	2	0	0	1.048,D	0.985,D	
94	DOCK10_c.4785delT p.Phe1595LeufsTer20 chr2:224,797,005	Frameshift	A3	not	0	0	N/A	N/A	
95	ITGB1BP1_c.241G>A p.Gly81Ser chr2:9,412,316	Missense	B1	not	0.0000012	0	−1.408,B	0.051,B	Low in mammals
	ARPC1B_c.826G>A p.Ala276Thr chr7:99,392,713	AR missen	B1	not	0.000049	0	−0.356,B	0.001,B	
96	MYH7_c.4048G>A p.Glu1350Lys chr14:23,418,331	Missense	B0	not	0	0	0.848,D	0.997,D	
	SEC16A_c.2626G>A p.Gly876Ser chr9:136,474,990	Missense	A3	not	0.000033		−0.479,B	0.003,B	Low in mammals
	AASDH_c.50G>C p.Arg17Thr chr4:56,384,250	Missense	B2	not	0	0	−1.357,B	0.008,B	
97	DLG2_c.1657T>C p.Phe553Leu chr11:83,833,679	Missense	A2	2	0	0	1.199,D	1.0,D	
	OPRK1_c.377T>C p.Phe126Ser chr8:53,234,992	Missense	A3	not	0.0000062	0	1.199,D	0.977,D	
	MT-RNR2 m.2647G>A; heteroplasmy: subject 26%, mother 0%	mtDNA	A1	1	0	0.00001772	N/A	N/A	High in mammals
98									
99	FAM178B_c.1207G>A p.Glu403Lys chr2:96,923,570	Missense	B2	not	0.0000078		0.002,P	0.001,B	Low in mammals
	NKX1-1_c.1078A>T p.Thr360Ser chr4:1,403,201	Missense	A3	not	0.000017		0.804,D	0.963,D	
	PPFIA2_c.343G>A p.Glu115Lys chr12:81,457,827	Missense	B0	not	0	0	0.935,D	0.998,D	
100	EP300_c.2747C>T p.Ser916Leu chr22:41,150,128	Missense	A1	1	0	0	0.892,D	1.0,D	
	CELSR2_c.1277G>A p.Arg426Gln chr1:109,251,356	Missense	A3	not	0.0000019	0	1.048,D	0.812,B	High in mammals

Note: Every variant detected, genome-wide, that alters the amino acid code of any genes is shown and evaluated in this table. Light green and light orange background indicate data that met, and failed to meet, our scoring criteria, respectively. Light green background in column 2 indicates that all criteria were satisfied for designation as a Principal Diagnostic Variant (PDV), and thus likely to be disease associated in a substantial manner. Light green background in column 1 indicates that the subject has at least one PDV. Light gray background in column 2 indicates genes that are well-established to segregate in an autosomal recessive manner, without clear dominant inheritance being demonstrated (^5^ below is an exception). Yellow background denotes when the variant’s prevalence is >1/10 K yet the variant is widely considered to be Pathogenic/disease-associated, and was designated as a PDV. Although sequencing coverage was inadequate to firmly establish mosaicism and the mosaic proportions, the red font in columns 2 and 3 indicates variants found at substantially less than 50% (relative to coverage) such that they likely are mosaic. The red font in columns 8 and 9 indicates values corresponding to at least moderate conservation, roughly corresponding to conservation through at least mammals. ^1^ Based on our criteria explained in the Subjects and Methods section. ^2^ The proportion of alleles in the general population with that variant per gnomAD. For rare variants, the number can be doubled to closely approximate the proportion of people with that variant. For most variant types, #1 and 2 are the allelic frequencies from genomes and exomes, respectively. For mtDNA variants, #1 and 2 are the allelic frequencies in homoplasmic and heteroplasmic forms, respectively. ^3^ When the computer algorithms for evolutionary conservation (PhyloP and PhastCons) did not agree, conservation was determined manually by the University of California, Santa Cruz Genome Browser (UCSC-GB). ^4^ The variant is present on the mitochondrial DNA (mtDNA) regardless of variant type. ^5^ A de novo variant in an established recessive gene was designated as a PDV because another rare and conserved variant (shown in the row below) was also identified in that gene. The variants are too distant from each other for phase to be established. ^6^ Splice site computer algorithmic predictions were not available, yet the nucleotide was found to be highly conserved via the UCSC-GB. ^7^ Per Online Mendelian Inheritance in Man (OMIM), loss-of-function variants in this gene are recessive in inheritance.

**Table 2:** Primary Diagnostic Variants (PDVs) that were and were not listed in the official laboratory report, with information regarding protein function.

Subject # ^1^	Gene(s) with De Novo Variant ^2^	Designation in Report ^3^	Disease Status ^4^	Case Reports (Individuals, Families, Publications) ^5^	NDD per HGMD ^6^	Protein Function ^7^	Ion Transport	Red- Ox Mito	Imm-une	Ubiquitin	Synapse	Express	Neuro Gen	CytoSkel	Cell–Cell	Signaling	Danger
3	*ANK2*	Other Variants	Known	36, 30, 10	64	Spectrin–actin cytoskeleton											
10	*ASXL1*	Positive	Known	135, 134, 121	6	Gene silencing, developmental roles											
11	*SLC6A1*	Other Variants	Known	18, 18, 15	53	GABA transporter											
11	*DSCAM*	Other Variants	Very rare	7, 6, 6	38	Neural cell adhesion molecule											
16	*MAST3*	Other Variants	Known	13, 7, 2	1	Serine/threonine kinase											
17	*SHANK3*	Positive	Known	188, 206, 64	91	Synaptic scaffolding protein											
32	*KDM5B*	Uncertain	Very rare	4, 3, 3	31	Demethylase, gene repression											
33	*APBB1*	Likely Negative	Novel	0, 0, 0	1	Transcription coregulator											
34	*ASPM*	Other Variants	Known	85, 75, 24	13	Mitotic spindle function in embryonic neuroblasts											
37	*HNRNPDL*	Negative	Known	118, 78, 24	0	mRNA splicing and nuclear export											
40	10q21.3q22.2x1 (65,164,362–74,517,047) 9.35 Mb deletion [>170/many/3 genes]	Positive	Known	ADK: 50, 45, 36 CTNNA3: 4, 4, 4 AGAP5: 2, 1, 1	ADK: 3 CTNNA3: 30 AGAP5: Not reported	ADK (51%): Adenosine kinase, regulator of extracellular and intracellular adenine/adenosine; anti-inflammatory agents CTNNA3: Catenin family, cell–cell adhesion; roles in blood–brain barrier and immune cell transmigration AGAP5: Possibly GTPase activator											
41	*CLCN4*	Positive	Known	62, 25, 8	18	Voltage-gated chloride channel											
44	16p11.2p11.2x1 (29,520,000–30,226,500) 706.50 kb deletion [39/31/5]	Positive	Very rare	CORO1A: 7, 5, 5 SEZ6L2: 9, 9, 6 KCTD13: 0, 0, 0 TAOK2: 1, 1, 1 MAPK3: 9, 9, 5	CORO1A: 1 SEZ6L2: 1 KCTD13: 11 TAOK2: 3 MAPK3: 5	CORO1A: possibly cell cycle progression, signal transduction, apoptosis, and gene regulation SEZ6L2: May contribute to specialized endoplasmic reticulum functions in neurons KCTD13: ubiquitin-dependent protein catabolic process, signal transduction TAOK2: focal adhesion assembly, intracellular signal transduction MAPK3: Kinase, signaling cascade regulating cellular processes including differentiation											
73	8p23.3p23.1x1 (163,500–7,383,000) 7.22 Mb deletion [88/26/5]	Positive	Known	CLN8: 65, 52, 33 ARHGEF10: 4, 4, 4 MCPH1: 38, 22, 19 DLGAP2: 7, 7, 4 CSMD1: 10, 8, 8	CLN8: 4 ARHGEF10: 6 MCPH1: 10 DLGAP2: 17 CSMD1: 27	CLN8: Possibly lipid related, neuronal differentiation, protection against cell death ARHGEF10: Guanine nucleotide exchange; possibly role in neural morphogenesis MCPH1: DNA damage response protein, G2/M checkpoint arrest DLGAP2: Synapse organization and signaling in neuronal cells CSMD1: Likely involved in learning or memory											
97	*DLG2*	Supplementary	Very rare	6, 4, 3	11	Membrane-associated guanylate kinase, scaffold for the clustering of receptors, ion channels, and associated signaling proteins											
97	*OPRK1*	Supplementary	Known	2241, 546, 2	0	Opioid receptor											
100	*EP300*	Uncertain	Known	316, 313, 85	9	Histone acetyltransferase											
1	*SLC12A1*		Known	57, 51, 30	0	Na-K-Cl cotransporter											
2	*MYLK*		Known	117, 117, 8	0	Myosin light chain kinase											
3	*MYOSB*		Known	18, 7, 4	Not reported	Myoglobin											
5	*UNKL*		Novel	0, 0, 0	0	Ubiquitination											
8	*IQGAP2*		Very rare	1, 1, 1	2	GTPase binding, interacts with cytoskeleton, cell adhesion, and signaling molecules to regulate cell morphology and motility											
10	*APLP1*		Very rare	7, 2, 2	1	Transcriptional activator, synaptic maturation											
11	*NPAS3*		Very rare	4, 3, 3	2	Transcription factor, neurogenesis											
13	*EFR3B*		Novel	0, 0, 0	1	Localize phosphatidylinositol 4-kinase to the plasma membrane											
14	*KIDINS220*		Known	15, 12, 12	4	Controls neuronal cell survival, differentiation into exons and dendrites, and synaptic plasticity; interacts with membrane, cytosolic signaling, and cytoskeletal components											
14	*RIMS1*		Very rare	6, 5, 5	13	Regulates synaptic vesicle exocytosis, regulates voltage-gated calcium channels during neurotransmitter and insulin release											
16	*SH3RF1*		Novel	0, 0, 0	Not reported	E3 ubiquitin ligase, cell death response, calcium homeostasis											
19	Xp11.4p11.4x2 (37,818,872–37,842,030) 23.16 kb intragenic 2-copy-duplication (on X-chromosome in XY male) [1/1/0]		Very rare	DYNLT3: 1, 1, 1	DYNLT3: Not reported	DYNLT3 (79%): A dynein light chain—a motor protein—involved in the intracellular retrograde motility of vesicles and organelles along microtubules; transcriptional modulator											
21	19q13.33q13.33x3 (50,409,797–50,476,028) 66.23 kb 3-copy-duplication of 5 genes, incl 35% of *POLD1* [4/4/0]		Known	POLD1: 40, 34, 30 FAM71E1: 0, 0, 0 SPIB: 0, 0, 0 MYBPC2: 0, 0, 0	POLD1: 0 FAM71E1: 0 SPIB: 0 MYBPC2: 0	POLD1 (35%): Catalytic subunit of DNA polymerase delta; plays a critical role in DNA replication and repair FAM71E1 (69%): Innate immune response SPIB: Transcriptional activator, acts as a lymphoid-specific enhancer MYBPC2: Modifies the activity of actin-activated myosin ATPase											
22	*ABCB6*		Known	79, 4, 14	1	Heavy metal importer, mitochondrial porphyrin uptake											
24	*ARHGEF2*		Very rare	2, 2, 2	2	Rho GTPase, transcriptional factor binding; involvement in cell motility and polarization, dendritic spine morphology, antigen presentation, innate immune response, cell cycle regulation, and microtubule stability											
25	*COL6A3*		Known	609, 590, 41	1	Alpha-3 chain of type VI collagen											
25	*JPH3*		Known	13, 11, 7	1	Junctional complexes between the plasma membrane and endoplasmic reticulum, mediates cross talk between the cell surface and intracellular ion channels.											
27	*PLEKHH2*		Known	17, 17, 7	0	Predicted to enable actin binding activity, including cytoskeleton											
31	*AQP2*		Known	88, 61, 50	0	Aquaporin-2 water channel prominent in renal-collecting tubules											
33	*TMEFF1*		Very rare	1, 1, 1	0	Blocks viruses from entering neurons											
34	*CBARP*		Novel	0, 0, 0	Not reported	Regulation of calcium-ion-dependent exocytosis and voltage-gated calcium channel activity											
39	9p22.3p22.3x4 (15,405655–15,517,446) 111.79 kb 4-copy-duplication [3/2/0]		Known	SNAPC3: 0, 0, 0 PSIP1: 10, 10, 7	SNAPC3: 1 PSIP1: Not reported	SNAPC3: Transcription of both RNA polymerase II and III small-nuclear RNA genes PSIP1: Transcriptional coactivator involved in neuroepithelial stem cell differentiation and neurogenesis											
46	*CPVL*		Novel	0, 0, 0	0	Carboxypeptidase likely involved in lysosomal phagocytosis, the inflammatory protease cascade, and antigen presentation											
49	*TTN*		Known	215, 165, 116	14	Assembly and functioning of cardiac and striated myocyte											
50	*TTN*		Known	215, 165, 116	14	Assembly and functioning of cardiac and striated myocytes											
50	*MT-CYB*		Known	21, 21, 11	Not reported	mtDNA-encoded subunit of respiratory complex III											
51	*NNAT*		Very rare	2, 2, 1	Not reported	May regulate ion channels during brain development											
52	*MLXIPL*		Novel	0, 0, 0	0	Transcription factor for triglyceride synthesis genes											
52	*SLC4A5*		Novel	0, 0, 0	Not reported	Sodium bicarbonate cotransporter involved in intracellular pH regulation											
54	*ARHGAP8*		Novel	0, 0, 0	0	GTPase activator for the Rho-type GTPases. Involved in signaling pathways that regulate cell processes involved in cytoskeletal changes											
54	*GDI2*		Novel	0, 0, 0	Not reported	GDP-dissociation inhibitor, regulates intracellular membrane trafficking											
61	*KCNJ6*		Known	12, 12, 7	1	G protein-coupled inwardly rectifying potassium channel; may be involved in the regulation of insulin secretion by glucose and/or neurotransmitters											
61	*HECW1*		Novel	0, 0, 0	1	E3 ubiquitin protein ligase											
62	*USP20*		Novel	0, 0, 0	2	Deubiquitinating enzyme that plays a role in many cellular processes including autophagy, cellular antiviral response											
63	*ZNF865*		Novel	0, 0, 0	Not reported	Transcription factor											
63	*CNOT11*		Novel	0, 0, 0	1	Involved in nuclear-transcribed mRNA poly(A) tail shortening											
64	*ERF*		Known	32, 26, 26	1	Transcription factor; involved in development, apoptosis, and the regulation of telomerase											
66	*TRPV4*		Known	115, 85, 48	3	Ca^2+^-permeable, nonselective cation channel; regulation of systemic osmotic pressure											
66	*GPS1*		Novel	0, 0, 0	1	Suppresses G-protein and mitogen-activated signal transduction; essential regulator of the ubiquitin conjugation pathway											
68	*SETD1A*		Very rare	9, 7, 6	12	Histone lysine methyltransferase; involved in RNA processing and the DNA damage response											
71	*LRTM1*		Novel	0, 0, 0	Not reported	Axon guidance and negative chemotaxis, synapse assembly											
71	*RACK1*		Very rare	1, 1, 1	0	Regulation of signal transduction and vesicle-mediated transport; present in the phagocytic cup											
71	*AHNAK*		Very rare	3, 3, 2	6	Large structural scaffold protein involved in blood–brain barrier formation, cell structure and migration, cardiac calcium channel regulation, and neuronal cell differentiation											
72	*DMXL1*		Very rare	2, 2, 2	6	WD repeat protein, regulatory functions											
73	1q21.2q21.2x3 (150,442,000–150,519,000) 77.00 kb 3-copy-duplication [5/3/0]		Known	RPRD2: 0, 0, 0 TARS2: 3, 3, 3 ECM1: 84, 74, 59	RPRD2: 1 TARS2: 0 ECM1: 0	RPRD2 (88%): Involved in mRNA 3′-end processing TARS2: Mitochondrial aminoacyl-tRNA synthetase—mitochondrial translation ECM1: Negative regulator of endochondral bone mineralization											
74	*MAP4K1*		Novel	0, 0, 0	1	Serine/threonine-protein kinase; involved in several processes, including response to environmental stress, cell signaling, promoting apoptosis, hematopoietic lineage decisions and growth regulation, IL2 production											
77	*SLC4A8*		Novel	0, 0, 0	0	Sodium and bicarbonate cotransporter, important for pH regulation in neurons											
77	*ERAP1*		Very rare	3, 3, 1	0	Aminopeptidase involved in trimming HLA-class-I-binding precursors so that they can be presented on MHC class I molecules											
77	*PTPRS*		Known	13, 13, 8	0	Protein tyrosine phosphatase signaling protein involved in cell–cell interaction, primary axonogenesis, and axon guidance during embryogenesis; down-regulates activation of NF-kappa-B, TNF, interferon alpha, and interferon beta											
77	*TOX*		Known	603, 520, 1	0	Transcriptional regulator involved in chromatin assembly, transcription, and replication and may function to regulate T-cell development											
83	*STAB1*		Known	0, 0, 0	2	Roles in tissue homeostasis and remodeling, intracellular sorting and recycling, cell adhesion, and receptor scavenging; possible roles in angiogenesis, defense against bacterial infection											
87	*FMN1*		Novel	0, 0, 0	2	Roles in adherens junction formation and polymerization of linear actin cables; transcriptional activity											
89	*SHROOM2*		Very rare	3, 2, 1	0	Amiloride-sensitive sodium channel activity; regulates cytoskeletal organization and architecture of endothelial cells; roles in migration and angiogenesis											
91	*PTGFR*		Novel	0, 0, 0	0	G-protein-coupled receptor for prostaglandin F2-alpha, which activates a phosphatidylinositol–calcium second messenger system											
93	*HDLBP*		Very rare	4, 4, 4	2	Binds high-density lipoprotein; removes excess cholesterol levels in cells; binds RNA and can induce heterochromatin formation											
94	*DOCK10*		Novel	0, 0, 0	0	Guanosine nucleotide exchange factors for Rho GTPases; involved in cytokinesis; essential for dendritic spine morphogenesis in Purkinje cells and in hippocampal neurons; sustains B-cell lymphopoiesis											
96	*MYH7*		Known	797, 699, 155	1	Myosin heavy chain 7; interacts with actin for force generation; abundant in muscle but present ubiquitously including in brain											
99	*NKX1-1*		Novel	0, 0, 0	Not reported	Transcription factor homeobox protein; embryonic development											
99	*PPFIA2*		Novel	0, 0, 0	1	Liprin, a scaffold for the recruitment and anchoring of LAR family PTPases; binds to calcium-calmodulin-dependent serine protein kinase; important for axon guidance; scaffolding protein in the dendritic spines											
100	*CELSR2*		Very rare	1, 1, 1	2	Belongs to the flamingo subfamily of non-classic-type cadherins; likely a receptor involved in cell adhesion and receptor–ligand interactions; cell–cell signaling during nervous system formation											

Note: 1. All Primary Diagnostic Variants (PDVs) from Table 2 are shown herein. Light blue and light yellow backgrounds in column ^1^ indicate PDVs that were and were not listed in the laboratory report from Variantyx, respectively. For large copy number variants (CNVs), the number of involved genes is provided (total/coding/SFARI-listed). ^2^ For large CNVs incorporating > 3 genes, only SFARI-listed genes are shown in this table. ^3^ The text corresponds to the actual wording in the laboratory report in respect to that variant (with the exception of “Other Variants of Interest”), and the shading reflects the color on the report. ^4^ Cases are counted only if the phenotypic information on the individual is reported in at least as much detail as that displayed in the current Table 1. Novel/orange background: Novel disorder—the condition is unpublished in that no cases meeting that minimum standard are reported. Very rare/yellow background: 1 to 9 cases with phenotypic information are reported. Known/light green background: Ten or more cases with phenotypic information are reported. For CNVs with two or more genes affected, the designation refers to the highest designation (Known > Very Rare > Novel) among the genes listed. ^5^ The data shown refer to the number of individuals reported with minimal phenotypic information per the standard in #4 above. The first number refers to the total number of affected individuals reported with a presumed disease-associated variant in that gene. The second and third numbers refer to the number of families and publications, respectively. For example, one paper with affected siblings and a second paper with two unrelated affected individuals would count as 4 individuals in 3 families in 2 publications (4, 3, 2). ^6^ Cases of neurodevelopmental disorders (NDDs) listed in the Human Gene Mutation Database (HGMD) accessed on 17 June 2025 (https://www.hgmd.cf.ac.uk/ac/index.php). Listing in the HGMD alone with an NDD, but without published phenotypic information, did not qualify for inclusion in columns 4 and 5 of this Table. ^7^ Synopsis of the known functions of the protein, with an emphasis regarding the 11 mechanistic categories shown in this table. Information was generally obtained from GeneCards.com on 21 June 2025. Percentages in parentheses indicate the proportion of the coding region affected by the CNV; the entire gene is affected when not specified. For CNVs involving > 5 genes, only SFARI genes are listed in the Table. The 11 categories are (shown in the columns to the right): Red: ion transport; Orange: mitochondrial redox potential/energy metabolism/cell death responses; Yellow: immune system manifestations; Green: ubiquitin-related protein degradation pathway; Blue: synapse/neurotransmission-related; Purple: gene expression; Pink: neurogenesis/brain development; Light grey: cytoskeleton-related; Dark grey: cell–cell interactions including adhesion; Black: signaling pathways other than synaptic transmission; Brown: cell danger response.

**Table 3:** Small variants identified genome-wide in randomly selected subjects.

	A	B	C	D	E	F	G
1	**Variable or Statistics**	**De novo PDV**	**De novo**	**Inherited**	**Inherited**	**Inherited**	**Inherited**
2	***p*** **(Odds Ratio, 95% C.I.)**	**Missense**	**Silent**	**Missense**	**Silent**	**5′3′UTRs**	**Up/Downstream**
3	**Number of Subjects**	100	100	50	50	50	25
4	**Total Number of Variants**	43	19	7838	4526	14,334	8173
5	**Average Variants/Subject**	0.43	0.19	157	90.5	287	327
6	**Number of SFARI Variants**	11	4	696	506	1167	447
7	**SFARI Variants/Subject**	0.11	0.04	13.9	10.1	23.3	17.9
8	**% SFARI**	26.58%	21.05%	9.05%	11.09%	8.17%	5.43%
9	***p*** **/OR vs. de novo missense**	[-]	*0.73*	0.0004	0.0056	0.002	<0.0001
10	***p*** **/OR vs. de novo silent**	*0.73*	[-]	*0.0744*	0.18	*0.0506*	0.0068
11	***p*** **/OR vs. Missense**	0.0004 (3.4, 1.7–6.8)	*0.0744 (2.7, 0.01* *–* *8.3)*	[-]	<0.0001	*0.075*	<0.0001
12	***p*** **/OR vs. Silent**	0.0056 (2.6, 1.3–5.3)	*0.18 (2.1, 0.70* *–* *6.4)*	<0.0001 (1.3, 1.2–1.5)	[-]	<0.0001	<0.0001
13	***p*** **/OR vs. 5`3` UTRs**	0.0002 (3.8, 1.9–7.5)	*0.0506*	*0.075*	<0.0001 (1.4, 1.3–1.6)	[-]	<0.0001
14	***p*** **/OR vs. Up/Downstream**	<0.0001(5.8, 2.9–11)	0.0068 (4.6, 1.5–14)	<0.0001 (1.7, 1.5–1.9)	<0.0001 (2.2, 1.9–2.5)	<0.0001 (1.5, 1.4–1.7)	[-]

Note: Every variant was tallied genome-wide through all (20,000–23,000) genes for the variant types listed in the column headings for the number of subjects listed in row 1. *p*, probability; OR, odds ratio; C.I., confidence interval; UTRs, untranslated regions; SFARI, Simons Foundation Autism Research Initiative sfari.org (accessed throughout June 2025). Statistical analyses are per https://www.medcalc.org/calc/odds_ratio.php (accessed throughout July 2025). Formatting within cells is *p* (Odds Ratio, 95% C.I.). Figures in italic font are not statistically significant. Light blue background emphasizes that those figures are for de novo variants. Light green backgrounds indicate duplicate values also found to the left/below. Other colored backgrounds emphasize important comparisons discussed in the text. Copy number and mtDNA variants were excluded.

## Data Availability

The original contributions presented in this study are included in the article/Supplementary Material. Further inquiries can be directed to the corresponding author.

## Supplementary Materials

The following supporting information can be downloaded at: https://www.mdpi.com/article/10.3390/genes16091099/s1, Table S1: Clinical manifestations in our subjects.
