# Supplementary material for: De Novo Variants Predominate in Autism Spectrum Disorder
Source: Genes (Basel). 2025 Sep 17;16(9):1099. doi: 10.3390/genes16091099 (PMC12470000; doi:10.3390/genes16091099)
Supplement: Supplementary file 1 [file genes-16-01099-s001.zip › Boles et al 2025 Genes Table S1.pdf]

Table S1. Clinical manifestations in our subjects

Subject #	Age and Sex	Developmental	Verbal [1]	Seizure/EEG	Regression	Other Neuropsychiatric	Other Phenotypes
1	15M	Severe ID	No	Seizures	Multiple	OCD, anxiety	Abnormal feces, snoring
2	7M	Moderate ID	No	Seizures	One	Anxiety, receptive expressive language disorder	
3	6F	Moderate ID	No	None	One	Aggression	Sleep apnea, sickle cell anemia
4	8F	Moderate ID	No	Seizures	Multiple	Sensory integration disorder, aggression	Abnormal feces
5	7F	Severe ID	No	Abnormal EEG, no seizures	One	Feeding difficulties, receptive expressive language disorder, fine motor delay, gross motor delay	Sleep disorder, chronic constipation
6	22M	Moderate ID	Yes	Seizures, convulsions	Multiple	OCD, anxiety, speech delay	Unspecified disorder of immune mechanism, abnormal feces, sleep disorder, abnormal findings in stool
7	8F	Severe ID	Reduced	None	None	ADHD, receptive expressive language disorder	Snoring
8	7M	Moderate ID	Yes	None	None	ADHD, auditory processing disorder, receptive expressive language disorder, fine motor delay, gross motor delay	
9	6M	Severe ID	Reduced	Possible seizures	Multiple	Receptive expressive language disorder	
10	24F	Moderate ID	Reduced	Seizures, epilepsy	None	Speech delay, fine motor delay, gross motor delay	Abnormal feces, Bohring-Opitz syndrome
11	9M	Severe ID	Yes	Staring episodes	None	ADHD, fine motor delay, gross motor delay	Chronic constipation
12	18M	Severe ID	Reduced	Convulsions	Yes, chronic	OCD, anxiety, speech delay	Carnitine deficiency, hypogammaglobulinemia, abnormal feces
13	14F	Severe ID	Yes	Epilepsy with partial complex seizures		OCD, anxiety	
14	7M	Moderate ID	Reduced	None	One	ADHD, receptive expressive language disorder	
15	34M	Severe ID	No	Seizures, epilepsy	None	Speech delay	Sleep apnea, chronic constipation, chronic migraine headaches
16	10M	Not recorded	Reduced	Abnormal EEG	Multiple	OCD, avoidant-restrictive food intake disorder, aggression, self-injurious behavior	Sleep disorder, abnormal feces
17	9M	Severe ID	No	Seizures	Multiple		Phelan-McDermid syndrome
18	7M	Mild ID	Yes	None	One	OCD, avoidant-restrictive food intake disorder, ataxia, apraxia, receptive expressive language disorder, articulation delay	Hypotonia
19	7M	Severe ID	Reduced	Possible seizures	One	Apraxia, speech delay	Recurrent bacterial infection, abnormal feces
20	18M	Severe ID	Reduced	Seizures	Multiple	Fine motor delay, gross motor delay	Hypothyroidism, hypogammaglobulinemia, megacolon, constipation, sleep difficulties, fatigue, hypotonia
21	7M	Severe ID	No		Multiple		

22	7M	Moderate ID	Yes	Abnormal brain MRI, staring episodes	None		
23	14M	Severe ID	Reduced	None	One	ADHD, anxiety, obsession, hyperlexia	Environmental allergies
24	24F	Moderate ID	Reduced	Seizures, complex partial epilepsy			
25	8F	Severe ID	Reduced	None	None		Recurrent fevers
26	11M	Severe ID	No	Seizures, staring episodes, complex partial epilepsy	One		Constipation
27	12M	Severe ID	No	None	Multiple	Receptive expressive language disorder	Chronic constipation
28	7M	Moderate ID	Reduced	None	None	Tic disorder, feeding difficulties, receptive expressive language disorder, fine motor delay, gross motor delay, speech delay	Hypotonia
29	7M	Not Recorded	Yes	None	One		
30	5M	Moderate ID	Yes	Seizures, febrile convulsion, abnormal EEG	One	Receptive expressive language disorder	
31	17M	Moderate ID	Reduced	Seizures, convulsions	One	Anxiety	Unspecified disorder of immune mechanism, bacterial enteritis, balanitis, sleep apnea, headache, immunoglobulin G deficiency
32	18M	Moderate ID	Reduced	None	None	ADHD, anxiety, dysgraphia, speech delay, fine motor delay, gross motor delay	CIDP
33	12M	Moderate ID	Reduced	Seizures, epilepsy	Multiple	ADHD, speech delay	Sleep disorder
34	6M	Severe ID	No	Febrile convulsion	One		Lead exposure, stomach pain
35	9M	Moderate ID	Yes	Unresponsive episode	None	ADHD, fine motor delay, gross motor delay	Abnormal feces, hypogammaglobulinemia
36	4M	Moderate ID	No	Convulsion	One	Speech delay	Complex IV deficiency
37	14M	Severe ID		Seizures		Aggression	
38	8F	Severe ID	Reduced	None	None	ADHD, receptive expressive language disorder, speech delay	Cyclic vomiting syndrome, fatigue, mitochondrial cytopathy
39	24M	Severe ID	Reduced	Seizures, epileptiform attacks	Multiple	ADHD, PANDAS, insomnia, speech delay	Perennial allergic rhinitis, enterocolitis, abnormal feces, abdominal pain
40	22M	Mild ID	Yes	Abnormal EEG	None	Speech delay, fine motor delay, gross motor delay	Abnormal feces
41	12M	Moderate ID	Reduced	None	Multiple	ADHD, receptive expressive language disorder, speech delay, fine motor delay, gross motor delay	
42	7M	Severe ID	No	None	One	ADHD, speech delay, receptive expressive language disorder	Snoring, intestinal dysbiosis
43	7M	Normal	Reduced	None	None	Tics, ADD, hyperactivity, impulsivity, apraxia, speech delay, fine motor delay	Hypotonia, GERD

44	8M	Not recorded	Yes	Febrile seizures	None	Apraxia, sensory integration disorder, learning disability	Abnormal feces, failure to thrive, sleep apnea
45	21M	Moderate ID	Yes	None	None	ADHD, Asperger syndrome	
46	19M	Moderate ID	No	Seizures, convulsions	Multiple	Insomnia, apraxia, speech delay	Abnormal feces, environmental allergies, malabsorption
47	8M	Severe ID	Yes	Staring episodes	Multiple	Auditory processing disorder, hyperlexia, social communication disorder, speech delay, fine motor delay, gross motor delay	
48	6M	Severe ID	No	Staring episodes, possible febrile convulsion, abnormal EEG	One	Anxiety	
49	7M	Severe ID	No	None	Multiple	Apraxia, attention and contraction deficit, speech delay, fine motor delay, gross motor delay	Constipation
50	16M	Severe ID	Reduced	Seizures, generalized epilepsy, convulsions	One		Abnormal feces, obesity, stage 2 hypertension
51	10M	Severe ID	No	Febrile seizures, transient alteration of awareness	None	ADHD, receptive expressive language disorder, speech delay, fine motor delay, gross motor delay	
52	6M	Severe ID	No	Possible seizures, abnormal EEG			PTEN gene mutation
53	5M	Moderate ID	Yes	None	None	Receptive expressive language disorder, speech delay, fine motor delay, gross motor delay	
54	7M	Severe ID	No	None	One	ADHD, receptive expressive language disorder, speech delay	
55	7M	Severe ID	Reduced	Staring episodes	Multiple	Motor apraxia, receptive expressive language disorder, speech delay, fine motor delay	Complex IV deficiency
56	5F	Severe ID	Reduced	Staring episodes	None	Receptive expressive language disorder, feeding difficulties, speech delay	Neutropenia associated with autoimmune disease, chronic diarrhea
57	32M	Moderate ID	Yes	Seizures, epilepsy	Multiple	ADD, social communication disorder, speech delay	Non-restorative sleep, carnitine deficiency, eczema
58	8M	Severe ID	Reduced	Seizures	One	Receptive expressive language disorder, speech delay, fine motor delay	Immune disorder, long QT interval
59	22M	Severe ID	Yes	Staring episodes		Aggression, speech delay, fine motor delay, gross motor delay	
60	15M	Mild ID	Reduced	Seizures, convulsions	None	ADHD	Abnormal feces, hypothyroidism, bacterial enteritis, gastroparesis
61	5M	Severe ID	Reduced	Possible seizures	None	Apraxia, receptive expressive language	Hypotonia

						disorder	
62	16F	Severe ID	Yes	None	Multiple	Anxiety, binge eating	Autoimmune encephalitis, sleep breathing disorder, circadian sleep disorder, constipation
63	7M	Severe ID	Reduced	None	Multiple	Tics, avoidant-restrictive food intake disorder, apraxia, PANS/PANDAS, receptive expressive language disorder	Hypotonia
64	11F	Moderate ID	Reduced	None	None	Hyporeflexia, peripheral neuropathy, cerebellar stroke, cerebral edema, speech delay, gross motor delay, mixed receptive expressive language disorder	Hypotonia, abnormal urine oligosaccharides, adrenal insufficiency, cardiomyopathy, liver failure, decreased mitochondrial complex I activity, Reye-like episode, hypoglycemia, lactic acidosis, thrombocytopenia, strabismus, sleep disorder
65	12M	Severe ID	Yes	None	One	ADHD, tics, speech delay, fine motor delay, gross motor delay	Abnormal feces, hyperlipidemia, polyuria, non-restorative sleep
66	15M	Severe ID	Reduced	None		Mixed receptive expressive language disorder	
67	9F	Severe ID	Reduced	Possible seizures		Anxiety, auditory processing disorder, learning disorders, speech delay, fine motor delay, gross motor delay	
68	6M	Moderate ID	Reduced	None	One	Receptive expressive language disorder	Carnitine deficiency
69	6F	Moderate ID	Reduced	None	One	Receptive expressive language disorder, fine motor delay, gross motor delay	
70	6M	Mild ID	Reduced	None	Multiple	Sensory processing difficulty, receptive expressive language disorder	
71	6M	Moderate ID	No	Seizures	Multiple	Receptive expressive language disorder, fine motor delay, gross motor delay	
72	30M	Severe ID	Reduced	Seizures	One	Bipolar, tics, OCD, hypersomnia, catatonia, developmental coordination disorder, insomnia, receptive expressive language disorder, self-injurious behavior	Headache, vitamin D intoxication, stereopathy
73	21M	Severe ID	Reduced	Seizures, epileptic convulsions, epilepsy	One	Hyperlexia, apraxia, social communication disorder, speech delay, fine motor delay, gross motor delay	Hypotonia
74	8F	Severe ID	Reduced	Seizures, staring episodes, febrile convulsion, abnormal EEG, epilepsy	Multiple	ADHD, insomnia, receptive expressive language disorder, hyperactivity, fine motor delay, gross motor delay	Restless sleeper

75	12M	Moderate ID	Reduced	None	None	Anxiety, ADHD, dyslexia, learning disorders, aggression, speech delay, fine motor delay, gross motor delay	
76	10M	Severe ID	No	None	None	Anxiety, ADHD, gross motor delay	Folate metabolism disorder
77	15M	Severe ID	No	Seizures, epilepsy	One	Insomnia, receptive expressive language disorder, fine motor delay, gross motor delay	Short stature, failure to thrive, enteritis
78	9M	Mild ID	Yes	Seizures, epileptiform attacks	None	Sensory processing difficulty, stereotyped behavior	Fecal abnormalities
79	5M	Moderate ID	No	None	Multiple	Receptive expressive language disorder	
80	11M	Severe ID	Yes	None	None	Aggression, irritability, anxiety, hyperactivity, impulsivity, self-injurious behavior	Appendicular hypotonia, positive folate receptor autoantibody test, sleep disorder, DNA depletion syndrome
81	4M	Normal	No	None	One	Receptive expressive language disorder	Intestinal dysbiosis, mitochondrial cytopathy
82	7M	Moderate ID	Yes	Seizures, epileptiform attacks	None	Tics, insomnia, Asperger syndrome, visual processing disorder	Abnormal feces, snoring, recurrent bacterial infection, primary immune deficiency disorder, strabismus
83	13M	Severe ID	Reduced	Possible seizures	Multiple	Receptive expressive language disorder	Constipation, carnitine deficiency
84	5M	Mild ID	No	None	Multiple	Speech delay	
85	8M	Severe ID	Reduced	Possible seizures	Multiple	ADHD, tics, receptive expressive language disorder	
86	14M	Moderate ID	Reduced	None	One	Apraxia, receptive expressive language disorder	
87	20M	Severe ID	Reduced	None	None	OCD, sensory integration dysfunction, developmental coordination disorder, receptive expressive language disorder	
88	7M	Mild ID	Yes	None	None	ADHD, receptive expressive language disorder, fine motor delay, gross motor delay	Failure to thrive
89	12F	Severe ID	Reduced	Epileptiform activity, staring episodes	Multiple	Social communication disorder	Abnormal feces, mitochondrial cytopathy
90	33F	Not recorded	Yes	None	One	OCD, anxiety, sensory integration disorder, learning disability, self-injurious behaviors	
91	17F	Severe ID	Reduced	None	One	Anxiety, OCD, speech delay, fine motor delay, gross motor delay	Chronic constipation
92	40F	Normal					Mitochondrial cytopathy, sleep arousal disorder, G6PD deficiency
93	12M	Severe ID	Yes	Staring episodes	One	Tics, ADHD, Tourette syndrome, Tic de Guinon	Prematurity
94	13M	Moderate ID	No	Possible seizures	One	Receptive expressive language disorder, fine motor delay	Genetic defect (DOCK10)

95	7M	Severe ID	Reduced	None	One	Speech delay, fine motor delay, gross motor delay	Constipation, complex IV deficiency, hypotonia
96	8M	Severe ID	Reduced	None	None	Tics, receptive expressive language disorder, obsessive behavior	Recurrent viral infection
97	26M	Moderate ID	No	Seizures	Multiple	OCD, anxiety, ADHD, dyspraxia, auditory processing disorder, apraxia, impulsive, aggression, self-injurious behavior, speech delay	Abnormal feces, carnitine deficiency, hypothyroid, ANA positive, bilateral leg pain, hypotonia
98	8M	Not Recorded	Reduced	Episodes of disrupted behavior and laughing, seizure-like, abnormal EEG	One	Aggression, ADD, speech delay, anxiety, hyperactivity, stereotypy, receptive expressive language disorder	Abnormal feces, non-restorative speech, folate receptor autoantibody test positive
99	4M	Severe ID	No	Staring episodes	One	Receptive expressive language disorder, fine motor delay	
100	12F	Severe ID	No	Staring episodes	One	Receptive expressive language disorder, developmental regression	Disorder of initiating and maintaining sleep, abdominal bloating, mitochondrial complex 1 deficiency

Table S1 Notes: EEG = electroencephalogram, ID = intellectual disability, OCD = obsessive–compulsive disorder, ADD/ADHD= attention deficit disorder without/with hyperactivity, POTS = postural orthostatic tachycardia syndrome, CIDP = chronic inflammatory demyelinating polyneuropathy, [1] Reduced speech is part of the diagnostic criteria for autism; cases were flagged with light green background only when expressive speech was essentially absent. Light blue highlighting in the penultimate column of Table 1 is for tics (9 cases, 9%) as a marker for potential PANS/PANDAS, as some level of obsessive traits is so common in autism that OCD is difficult to differentiate from background. This is an incomplete listing limited to selected manifestations recorded in the clinical records available.
